# A Review of Chinese Species of the Genus *Oides* Weber, 1801 (Coleoptera: Chrysomelidae: Galerucinae)

**DOI:** 10.3390/insects15020114

**Published:** 2024-02-05

**Authors:** Meixia Yang, Jian Shen, Changping Ding, Xingke Yang

**Affiliations:** 1 Shaanxi Institute of Zoology, Xi’an 710000, China; ymeixia2013@163.com (M.Y.);; 2 Key Laboratory of Zoological Systematics and Evolution, Institute of Zoology, Chinese Academy of Sciences, Beijing 100101, China

**Keywords:** leaf beetles, new species, *Oides*, morphology, taxonomy

## Abstract

**Simple Summary:**

The genus *Oides* belongs to the subfamily Galerucinae and is widely distributed in the Old World. In this study, Chinese species of the genus *Oides* were revised based on comparative morphological characteristics. In total, seven new species and a new record in China are described. A key to all the Chinese *Oides* species is provided.

**Abstract:**

In this study, 25 species of *Oides* Weber from China were reviewed. Among them, the following seven new species are described: *Oides angusta* **sp. nov.**, *O. cystoprocessa* **sp. nov.**, *O. paraboreri* **sp. nov.**, *O. parabowringii* **sp. nov.**, *O. parathibettana* **sp. nov.**, *O. shimenensis* **sp. nov.**, and *O. yunnanensis* **sp. nov.**; *Oides innocua* Gahan has been recorded in China for the first time. A key to all the Chinese *Oides* species is provided.

## 1. Introduction

The genus *Oides* Weber, belonging to Galerucinae (Coleoptera: Chrysomelidae), was established by Weber in 1801. *Chrysomela bipunctata* Fabricius, 1781was designated as the type species of the genus *Oides* by Weise, 1924. This genus is widespread in the Old World and is the most diverse in tropical regions [1]. Yang *et al.* (2015) systematically organized Chinese leaf beetles and recorded 15 *Oides* species that were distributed in China [2]. Lee & Beenen (2017) revised the genus *Oides* distributed in the Palaearctic and Oriental regions by examining all extant and valid types and concluded as follows: (1) The specimens coming from China, previously reported as *O. duporti* Laboissière, 1919, really belonged to *O. leucomelaena* Weise, 1922; (2) *O. andrewesi* Jacoby and *O. maculata* (Olivier, 1807) were not yet distributed in China; (3) The distribution of *O. flava* (Olivier, 1807) and *O. multimaculata* Pic, 1928 in China needed confirmation due to the lack of examination of specimens collected from China; (4) *O. gyironga* Chen *et* Jiang, 1981 was a synonym of *O. scutellata* (Hope, 1831), and *O. chinensis* Weise, 1922 was a synonym of *O. tarsata* (Baly, 1865); (5) *O. epipleuralis* Laboissière, 1929, *O. laticlava* (Fairmaire, 1889), *O. palleata* (Fabricius, 1781), and *O. thibettana* Jacoby, 1900 were added to the *Oides* species that were distributed in China [1]. Thus far, 15 species have been known in China, including 2 species whose distribution in China is still in doubt.

In this study, Chinese *Oides* specimens conserved in the Institute of Zoology, Chinese Academy of Sciences, Beijing, China (IZAS) and Northwest Agriculture and Forest University (NWAFU) were examined. We recorded seven new species and clarified the distribution of *O. duporti* Laboissière, 1919, *O. maculata* (Olivier, 1807), and *O. multimaculata* Pic, 1928 in China.

## 2. Materials and Methods

In this study, the type specimens of the new species and most of the specimens examined are conserved in the Institute of Zoology, Chinese Academy of Sciences, Beijing, China (IZAS). Examined specimens from Northwest Agriculture and Forest University were marked by NWAFU. The specimens were mainly collected from provinces in the Chinese mainland.

The extraction of genitalia was performed under a Nikon SMZ745 stereomicroscope. The specimen was placed under the stereomicroscope with the ventral side facing up. The tip of a needle was inserted into the thoracoabdominal joint; then, the entire abdomen was gently pried and removed. The whole abdomen was boiled in a 10% NaOH solution for about 3–5 min (the specific time depended on the ossification degree of the specimen), and it was then taken out when the muscle dissolved completely. The treated abdomen was rinsed in distilled water and placed in a clean petri dish with the ventral side facing up, followed by extraction of the external genitalia with tweezers. The abdomen and external genitalia were stored together in a micro-centrifuge tube with glycerin, and the centrifuge tube was stored with the needle specimen on the same needle.

Photographs of the habitus and genitals were taken with a Leica DFC450 micro digital imaging system (CCD), attached to a Leica M205C microscope. All figures were edited using Adobe Photoshop CS 6.0.

## 3. Results

### 3.1. Taxonomy

*Oides* Weber, 1801

*Oides* Weber, 1801: 26 [3]. Type species: *Chrysomela bipunctata* Fabricius, 1781, designated by Weise, 1924.

*Adorium* Fabricius, 1801: 409 [4]. Type species: *Chrysomela bipunctata* Fabricius, 1781, designated by Duponchel, 1841. Synonymized by Harold, 1876: 3555 [5].

*Isosoma* Billberg, 1820: 56 [6]. Type species: *Chrysomela concolor* Fabricius, 1781, designated by Barber, 1947. Synonymized by Barber, 1947: 153 [7].

*Ochralea* Chevrolat, 1836: 375 [8]. Type species: *Adorium flavum* Olivier, 1807. Synonymized by Beenen, 2010: 491 [9].

*Callipepla* Dejean, 1837: 399 [10]. Type species: *Adorium posticum* Boisduval, 1835, designated by Wilcox, 1971. Synonymized by Duponchel, 1843: 55 [11].

*Rhombopalpa* Chevrolat, 1837: 399 (ed. 2, p. 375) [10]. Type species: *Adorium decempunctatum* Billberg, 1808, by monotypy. Synonymized by Harold, 1876: 3555 [5].

*Galleruca* subgen. *Boisduvalia* Montrouzier, 1855: 72 [12]. Type species: *Galeruca* (*Boisduvalia*) *sexlineata* Montrouzier, 1855, designated by Wilcox, 1971. Synonymized by Harold, 1876: 3555 [5].

*Rhombopala*: Clark, 1865: 143 [error for *Rhombopalpa*] [13].

*Botanoctona* Fairmaire, 1877: 185 [14]. Type species: *Botanoctona pallidocincta* Fairmaire, 1877, by monotypy. Synonymized by Weise, 1924: 1 [15].

*Arorium* Fairmaire, 1887: 362 [error] [16].

**Generic Character:** Body length 7.4–17.8 mm. Antennae shorter than body length; antennomere II shortest, about 1.5× shorter than III. Pronotum wider than long, with sharp front angles and rounded back angles, extended laterally; disc convex, with or without fine punctures; scutellum tongue-like. Elytra oval, middle widest, disc convex with punctures and reticulated microsculpture; epipleurae wide, 1/3 or 1/2 the width of the elytra, forming lateral margins of the elytron. Anterior coxal cavities open; the length of the first tarsal segment equal to the sum of about 2 + 3 segments; claw bifurcated. Apex of abdomen in male with a rectangular depression.

**Distribution:** Palaearctic, Indomalayan, Afrotropical, and Australian regions.

### 3.2. Species Descriptions

#### 3.2.1. *Oides angusta* **sp. nov.** (Figure 1A–I)

**Description. Male.** Length 9.5–13.0 mm, width 7.5–11.0 mm. **Female.** Length 13.0–14.5 mm, width 8.5–9.5 mm.

Body yellowish-brown. Antennomeres VIII–XI, metapleura and metasternum black; leg yellowish-brown, apex of tibia and ventral part of tarsus dark brown; abdomen yellowish-brown, each ventrite with one pair of black longitudinal spots. Antennae filiform, 3/5 the length of body; antennomere I dilated, II shortest, III longest, IV slightly shorter than III; V–VII subequal in length, slightly shorter than IV; VIII–XI subequal in length, slightly shorter than VII. Pronotum transverse, more than 2.0× wider than long; anterior margin concaved, front angles protruding anteriorly, back angles obtuse, disc with dense punctures. Scutellum tongue-like, smooth without punctures. Elytra oval, disc with dense punctures, diameter of puncture shorter than spacing between punctures; epipleurae narrower than 1/3 the width of elytra. Aedeagus well sclerotized, nearly parallel-sided; apex slightly enlarged in dorsal view, the dorsal apex processes on both sides lobate, terminal of apical processes on ventral side rounded; the aedeagus narrowed dorsally at basal 1/2 in lateral view. Female sternite VIII strongly sclerotized, apical margin wide, depressed medially, with dense setae. Gonocoxae U-shaped, paired gonocoxae connected by membrane, apical region widest, narrowed toward basal, apex with dense setae. Spermatheca strongly curved and hook-like, basal slender and slightly dilated with proximal end sclerotized; middle sclerotized, as wide as apex. Apical region strongly sclerotized and widely rounded.

**Holotype:** ♂, China, Hubei Prov., Shennongjia, 500 m, 3 June 1981, leg. Han Yinheng. **Paratypes:** China: 1♂, Henan Prov., Shangcheng Country, Suxianshi Town, Xihe Protection Station, 358.62 m, 7 October 2021, 31.73628° N, 115.54872° E, roadside, scrub, leg. Chenjun and Liang Hongbin; 1♂, Shaanxi Prov., Shangnan Country, 16 September 1982, hawkthorn, leg. Sun Yizhi (NWAFU); 1♂, Anhui Prov., Jinzhai Country, Tangjiahui Town, Jingangtai Village, Jingangtai, 485.18 m, 5 May 2021, 31.68366° N, 115.52624° E, tea garden, woods, leg. Zhao Kaidong and Zhu Xichao; 3♂1♀, Hunan Prov., Shimen Country, Hupingshan Town, Nanping Village, Maozhu River, 320 m, 13 October 2014, 30.6180° N, 110.9046° E, leg. Liang Hongbin; 1♀, Hunan Prov., Shimen Country, Hupingshan Town, Shangyanhe Village, 632 m, 16 October 2014, 30.0477° N, 110.9503° E, leg. Liang Hongbin; 1♂, Fujian Prov., Chong’an Country, Xing Village, Tongmuguan, 900–1150 m, 30 May 1960, leg. Zhang Yiran; 1♂, Sichuan Prov., Mt. Emei, Baoguo Temple, 550–750 m, 9 July 1957, leg. Lu Youcai; 1♂, Yunnan Prov., Yanjin Country, 1000 m, 19 June 1980, leg. Hu Zongli; 1♂, Yunnan Prov., Xishuangbanna, Menghun, 1200–1400 m, 18 May 1958, leg. Zhang Yiran; 2♂, Guizhou Prov., Guiyang City, Yeyatang, 1980, collector unknown.

**Figure 1:**
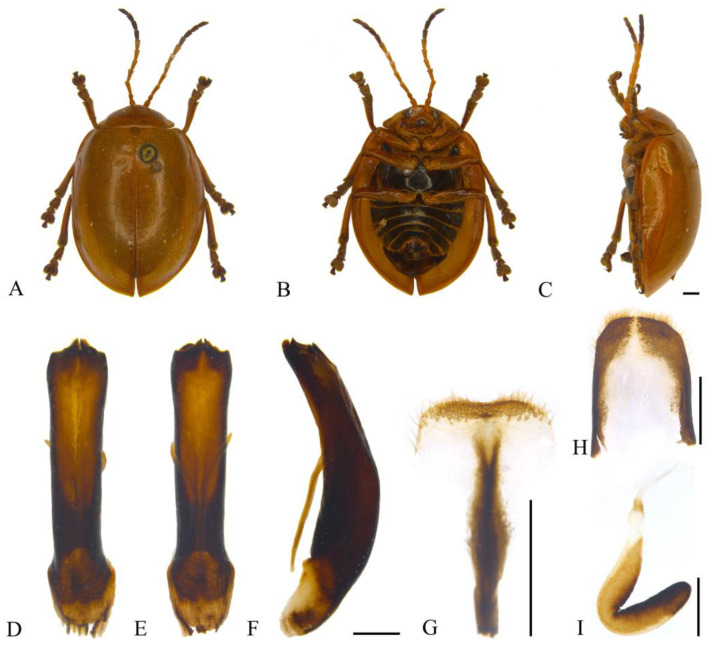
*Oides angusta* **sp. nov.** (**A**) Habitus, dorsal view; (**B**) habitus, ventral view; (**C**) habitus, lateral view; (**D**) aedeagus, dorsal view; (**E**) aedeagus, ventral view; (**F**) aedeagus, lateral view; (**G**) female sternite VIII; (**H**) gonocoxae; (**I**) spermatheca. Scale bars: 1 mm.

**Diagnosis.** The new species is similar to *Oides tarsata* (Baly), but antennomere III is longer than IV; epipleurae is narrower, and the morphology of male genitalia is completely different.

**Etymology.** The specific name is derived from the Latin word “*angustus*”*,* referring to the aedeagus constricts dorsally at the middle.

**Figure 2:**
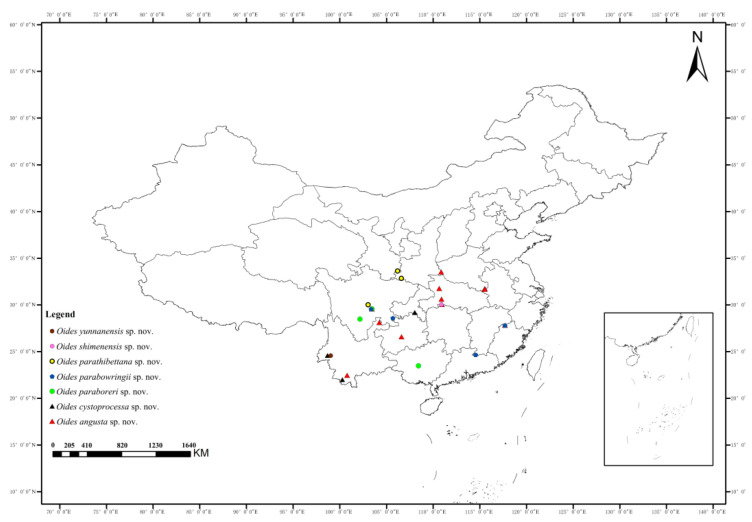
Distribution map of *Oides angusta* **sp. nov.**, *O. cystoprocessa* **sp. nov.**, *O. paraboreri* **sp. nov.**, *O. parabowringii* **sp. nov.**, *O. parathibettana* **sp. nov.**, *O. shimenensis* **sp. nov.** and *O. yunnanensis* **sp. nov.**

**Distribution.** China (Figure 2): Henan, Shaanxi, Anhui, Hubei, Hunan, Fujian, Sichuan, Guizhou, Yunnan.

#### 3.2.2. *Oides bowringii* (Baly, 1863) (Figure 3A–F)

*Adorium bowringii* Baly, 1863: 623 [17].

*Oides bowringii*: Harold, 1876: 3555 [5].

*Oides elegans* Laboissière, 1919: 161 [18]. Synonymized by Gressitt & Kimoto, 1963: 476 [19].

*Oides tonkinensis* Laboissière, 1929: 252 [20]. Synonymized by Kimoto, 1989: 36 [21].

**Specimens examined.** China: 1♂, Shaanxi Prov., Nanzheng Country, date and collector unknown; 1♀, Zhejiang Prov., Mt. Tianmu, 500–1000 m, 22 July 1973, leg. Yu Peiyu; 1♀, Hubei Prov., Xuanen Country, Xiaoguan, 1000 m, 4 August 1989, leg. Mai Guoqing; 1♀, Jiangxi Prov., Longnan Country, Mt. Jiulian, 9 June 1975, leg. Zhang Youwei; 1♀, Hunan Prov., Hengyang City, Nanyue, 1100 m, August 1963, collector unknown; 1♀, Hunan Prov., Yongshun Country, Shanmuhe State Forest Farm, 600 m, 9 August 1988, leg. Wang Shuyong; 1♀, Hunan Prov., Zhangjiajie City, 8 August 2001, collector unknown; 1♂, Fujian Prov., Chong’an Country, Xingcun Town, Tongmuguan, 800–1000 m, 21 July 1960, leg. Jiang Shengqiao; 1♀, Fujian Prov., Jiangle Country, Mt. Longqi, 650 m, 13 June 1991, leg. Yang Longlong; 1♂, Guangxi Prov., Longsheng Country, Sanmen Town, 300 m, 21 June 1963, leg. Shi Yongshan; 1♂, Guangxi Prov., Mt. Mao’er, 1150 m, 8 July 1985, leg. Liao Subai; 1♀, Guangxi Prov., Huaping, 12 July 1983, collector unknown; 1♀, Guangxi Prov., Baishou Country, 3 July 1952, collector unknown; 1♂, Sichuan Prov., Mt. Emei, Xixiangchi, 1800–2000 m, 12 August 1957, leg. Zhu Fuxing; 1♂, Sichuan Prov., Wenchuan Country, Yingxiu Town, 900 m, 1 August 1983, leg. Wang Shuyong; 1♀, Sichuan Prov., Wan Country, Wang’erbao, 1200 m, 10 August 1993, leg. Song Shimei; 1♂, Sichuan Prov., Jiangjin Country, 17 August 1979, collector unknown; 1♂, Guizhou Prov., Dasha River, Xiannvdong, 650–1150 m, 21–23 August 2004, leg. Yang Maofa; 1♂, Guizhou Prov., Jiangkou Country, Mt. Fanjing, 530 m, 12 July 1988, leg. Wang Shuyong; 1♂, Guizhou Prov., Maolan Town, Dongtang, 25 May 1998, leg. Song Qiongzhang; 1♂, Guizhou Prov., Jiangkou Country, Mt. Fanjing, 530 m, 12 July 1988, leg. Wang Shuyong; 1♂, Guizhou Prov., Mt. Leigong, 14 August 1988, leg. Li Yongkun; 1♂, Yunnan Prov., Pingbian Country, Mt. Dawei, 1350 m, 21 June 1956, leg. Bonfilov; 1♂, Yunnan Prov., Pingbian Country, Mt. Dawei, 1500 m, 17 June 1956, leg. Huang Keren; 1♂, Yunnan Prov., Pingbian Country, Mt. Dawei, 1350 m, 21 June 1956; 1♀, Yunnan Prov., Dongshan Country, No.12 Bridge to Dulongjiang, 27°42′54″ N, 98°30′8″ E, 2770 m, 30 April 2002, leg. Liang Hongbin, Ba Weidong and Li Xiangqian.

**Distribution.** China: Shaanxi, Gansu, Zhejiang, Hubei, Jiangxi, Hunan, Fujian, Guangdong, Hainan, Hongkong, Guangxi, Sichuan, Guizhou, Yunnan; Japan; Vietnam; Laos; Myanmar.

**Host plant. ***Schisandra chinensis* (Turczaninow).

**Figure 3:**
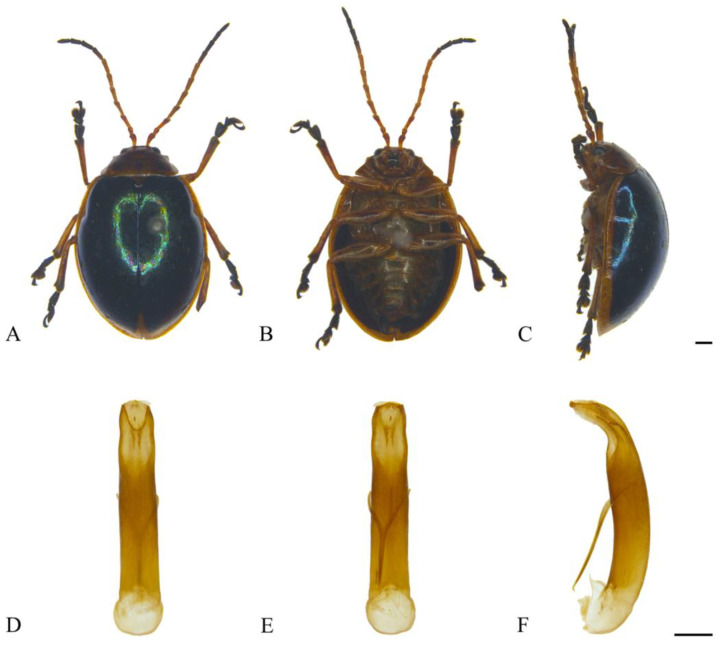
*Oides bowringii* (Baly) (♂). (**A**) Habitus, dorsal view; (**B**) habitus, ventral view; (**C**) habitus, lateral view; (**D**) aedeagus, dorsal view; (**E**) aedeagus, ventral view; (**F**) aedeagus, lateral view. Scale bars: 1 mm.

#### 3.2.3. *Oides coccinelloides* Gahan, 1891 (Figure 4A–F)

*Oides coccinelloides* Gahan, 1891: 458 [22].

**Specimens examined.** China: 1♀, Yunnan Prov., Ruili City, 1400 m, 6 June 1956, leg. Zhou Benshou; 1♂, Xizang, Motuo Country, Xirang Village, 800 m, 4 August 1974, leg. Huang Fusheng; 1♂, Xizang, Motuo Country, Pangxin Village, 1700 m, 19 June 1998, leg. Yao Jian; 1♀, Xizang, Motuo Country, Gandai, 2150 m, 25 June 1998, leg. Yao Jian; 1♀, Xizang, Motuo Country, Bangxin Village, 1300–1500 m, 11 July 1982, leg. Han Yinheng; 1♀, Xizang, Motuo Country, Beibeng Village, 800–1200 m, 10 June 1983, leg. Han Yinheng.

**Distribution.** China: Yunnan, Xizang; India; Myanmar.

**Figure 4:**
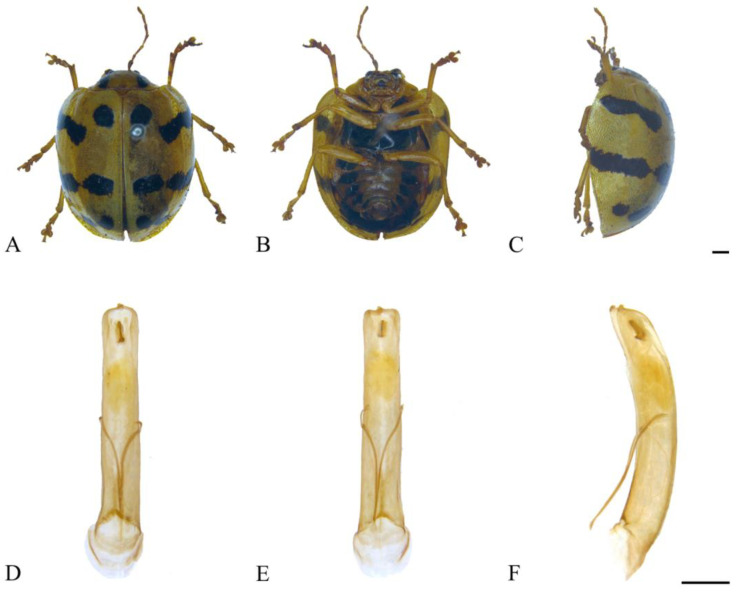
*Oides coccinelloides* Gahan (♂). (**A**) Habitus, dorsal view; (**B**) habitus, ventral view; (**C**) habitus, lateral view; (**D**) aedeagus, dorsal view; (**E**) aedeagus, ventral view; (**F**) aedeagus, lateral view. Scale bars: 1mm.

#### 3.2.4. *Oides cystoprocessa* **sp. nov.** (Figure 5A–H)

**Description. Male.** Length 10.5–11.5 mm, width 7.5–8.5 mm. **Female.** Length 11.0–11.5 mm, width 7.5–8.5 mm.

Body yellowish-brown. Antennomeres IX–XI, metapleura and metasternum black; leg yellowish-brown, conjunction of tibia and tarsus dark brown; abdomen yellowish-brown, each ventrite with one pair of black longitudinal spots. Antennae filiform, 3/5 the length of the body; antennomere I dilated, II shortest, III longest, IV distinctly shorter than III; V–VII subequal in length, slightly shorter than IV; VIII–XI subequal in length, slightly shorter than VII. Pronotum transverse, about 2.0× wider than long; anterior margin concaved, front angles protruding anteriorly, back angles obtuse, disc with scattered indistinct punctures. Scutellum tongue-like, smooth without punctures. Elytra oval, disc with dense punctures, diameter of puncture longer than spacing between punctures; epipleurae about 1/3 the width of elytra. Aedeagus well sclerotized, widening from about basal 1/2 to apex in dorsal view; dorsal apical processes sharp; everted endophallus developed in ventral view; apex slightly dilated in lateral view. Female sternite VIII strongly sclerotized, shield-like, medial area projecting anteriorly and erect, and apical margin truncate with dense setae. Gonocoxae reduced. Spermatheca strongly sclerotized and curved, hook-like; proximal end of basal sclerotized, distal end of basal membranous and constricted; middle and apical regions equal in width, surface wrinkled, and apex widely rounded.

**Holotype:** ♂, China, Yunnan Prov., Xishuangbanna, Mengzhe, 1200 m, 3 July 1958, leg. Wang Shuyong. **Paratypes:** China: 1♂1♀, Chongqing City, Pengshui Country, Mowei Mountain scenic zone, 1585 m, 23 May 2017, 29.1766° N, 108.0314° E, leg. Zhao Kaidong; 1♀, Chongqing City, Pengshui Country, Mowei Mountain scenic zone, 1585 m, 22–23 May 2017, 29.1766° N, 108.0314° E, leg. Song Zhishun; 1♀, Chongqing City, Pengshui Country, Mowei Mountain scenic zone, 1482 m, 22 May 2017, 29.1926° N, 108.0428° E, leg. Liu Hong and Zhao Kaidong; 1♂, Yunnan Prov., Longling Country, 1600 m, 20 May 1955, leg. Zhao Yi; 1♂, Yunnan Prov., Xishuangbanna, Mengzhe, 1150 m, 25 July 1958, leg. Wang Shuyong.

**Diagnosis.** The new species is similar to *Oides parathibettana* **sp. nov.**, but antennomeres IX-XI are black; everted endophallus of aedeagus is developed in lateral view, apex is acute; aedeagus constricts ventrally at basal 1/2.

**Etymology.** The specific name is derived from the Latin prefix “*cysto-*” and the Latin word “*processus*”, referring to the developed everted endophallus in ventral view.

**Distribution.** China (Figure 2): Chongqing, Yunnan.

**Figure 5:**
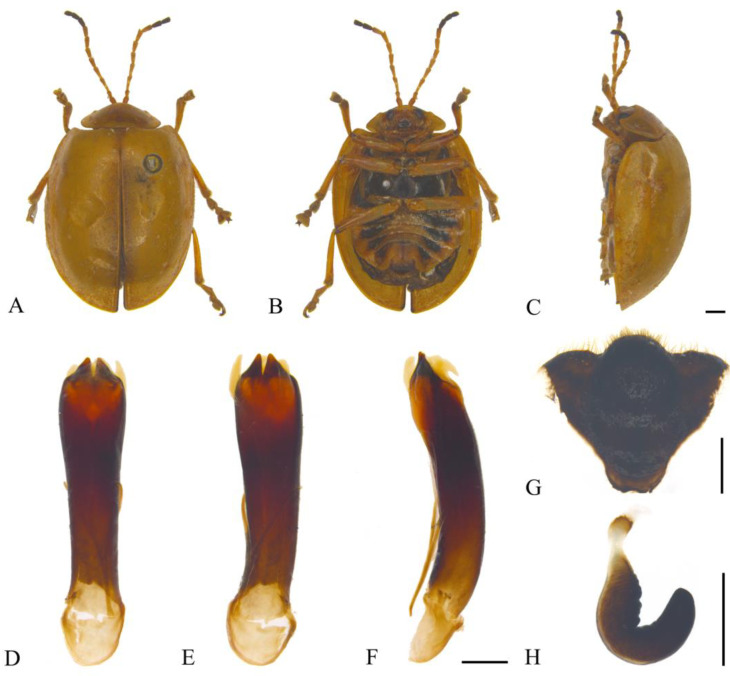
*Oides cystoprocessa* **sp. nov.** (**A**) Habitus, dorsal view; (**B**) habitus, ventral view; (**C**) habitus, lateral view; (**D**) aedeagus, dorsal view; (**E**) aedeagus, ventral view; (**F**) aedeagus, lateral view; (**G**) female sternite VIII; (**H**) spermatheca. Scale bars: 1 mm.

#### 3.2.5. *Oides decempunctata* (Billberg, 1808) (Figure 6A–F)

*Adorium decempunctata* Billberg, 1808: 230 [23].

*Oides decempunctata*: Harold, 1876: 3555 [5].

*Oides decemmaculata* Laboissière, 1927: 39 [24]. Synonymized by Kimoto, 1989: 36 [21].

*Solanophila gigantea* Roubal, 1929: 96 [25]. Synonymized by Roubal, 1931: 36 [26].

**Figure 6:**
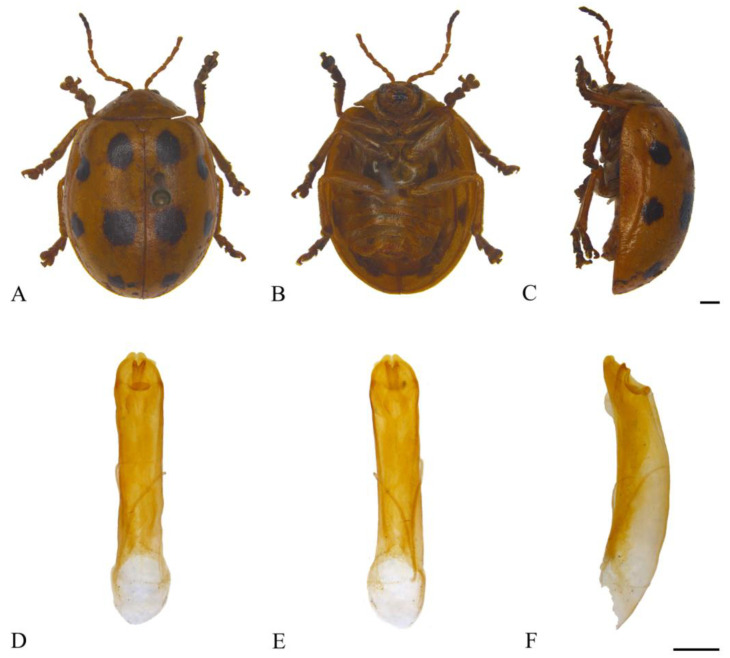
*Oides decempunctata* (Billberg) (♂). (**A**) Habitus, dorsal view; (**B**) habitus, ventral view; (**C**) habitus, lateral view; (**D**) aedeagus, dorsal view; (**E**) aedeagus, ventral view; (**F**) aedeagus, lateral view. Scale bars: 1 mm.

**Specimens examined.** China: 1♀, Liaoning Prov., Kuandian Country, 11 August 1984, leg. Jiangli; 2♂, Neimenggu, Zhemeng, Daqinggou, 30 July 1981, collector unknown; 3♂1♀, Beijing City, Haidian District, 22 August 1962, leg. Wang Shuyong; 3♀, Tianjin City, date and collector unknown; 2♂5♀, Shanxi Prov., Mt. Zhongtiao, 700 m, 29 July 1995, leg. Yang Xingke; 1♂, Shandong Prov., Jinan City, 28 September 1951, collector unknown; 1♂1♀, Henan Prov., Xin Country, 23 July 1985, leg. Han Yunfa; 1♂, Gansu Prov., Lanzhou City, 10 September 1933, leg. T.C. Ma; 3♂3♀, Shaanxi Prov., Changan Country, Mt. Cuihua, 22 August 1973, leg. Zhang Xuezhong; 1♀, Shaanxi Prov., Mt. Hua, 1000 m, 10 August 1972, leg. Wang Shuyong; 1♀, Shaanxi Prov., Shiquan Country, 3 August 1960, collector unknown; 1♀, Jiangsu Prov., Nanjing City, 16 July 1917, collector unknown; 1♀, Jiangsu Prov., Nantong City, Sanyu Town, 4 August 1955, collector unknown; 1♂1♀, Shanghai City, Songjiang District, Hengyun Mountain Ecological Forest, 121°8’33″ E, 31°3’29″ N, 15 July 2015, leg. Shen Min, Zhang Congcong and Zeng Mengman; 1♀, Anhui Prov., Yuexi Country, 25 June 1984, leg. Yu Fangbei; 1♀, Zhejiang Prov., Hangzhou City, 22 July 1933, collector unknown; 1♂2♀, Hubei Prov., Zigui Country, Maoping Town, 110 m, 3 September 1994, leg. Chen Jun; 1♀, Jiangxi Prov., Mt. Jiulian, 18 September 1979, leg. Chen Yuanqing; 1♂1♀, Jiangxi Prov., Nanchang City, 9 September 1979, leg. Yu Peiyu; 2♂1♀, Hunan Prov., Jishou City, 23 July 2001, leg. Ren Guodong; 1♂, Hunan Prov., Dayong Country, Zhushitou, 400 m, 21 August 1988, leg. Wang Shuyong; 1♀, Guangdong Prov., Lian Country, Luoyang Village, 22 July 1965, leg. Zhang Youwei; 1♂1♀, Hainan Prov., Xinglong, 15 June 1963, leg. Zhang Baolin; 6♂8♀, Guangxi Prov., Guilin City, Yanshan District, 200 m, 8 July 1963, leg. Wang Shuyong; 1♀, Guangxi Prov., Longzhou Country, Sanlian, 350 m, 13 June 2000, leg. Li Wenzhu; 1♂1♀, Guangxi Prov., Lipu, 25 July 1985, leg. Liao Subai; 1♂, Chongqing City, Beibei District, 11 September 1940, collector unknown; 1♂, Sichuan Prov., Tongjiang Country, 29 August 1980, leg. Zhang Jianjun; 7♂3♀, Guizhou Prov., Jiaokou Country, Mt. Fanjing, 530 m, 12 July 1988, leg. Yang Xingke; 1♀, Guizhou Prov., Tongren City, 19 October 1988, leg. Xu Huanli; 1♂, Yunnan Prov., Ruili City, 1300 m, 10 June 1956, leg. Huang Tianrong.

**Distribution.** China: Jilin, Inner Mongolia, Beijing, Tianjin, Hebei, Shanxi, Shandong, Henan, Shaanxi, Gansu, Shanghai, Jiangsu, Anhui, Zhejiang, Hubei, Jiangxi, Hunan, Fujian, Taiwan, Guangdong, Hainan, Guangxi, Chongqing, Sichuan, Guizhou, Yunnan; Russia; Korea; Vietnam; Laos; Thailand; Cambodia.

**Host plant.***Vitis* spp., Grape.

#### 3.2.6. *Oides duporti* Laboissière, 1919 (Figure 7A–F)

*Oides duporti* Laboissière, 1919: 160 [18].

**Figure 7:**
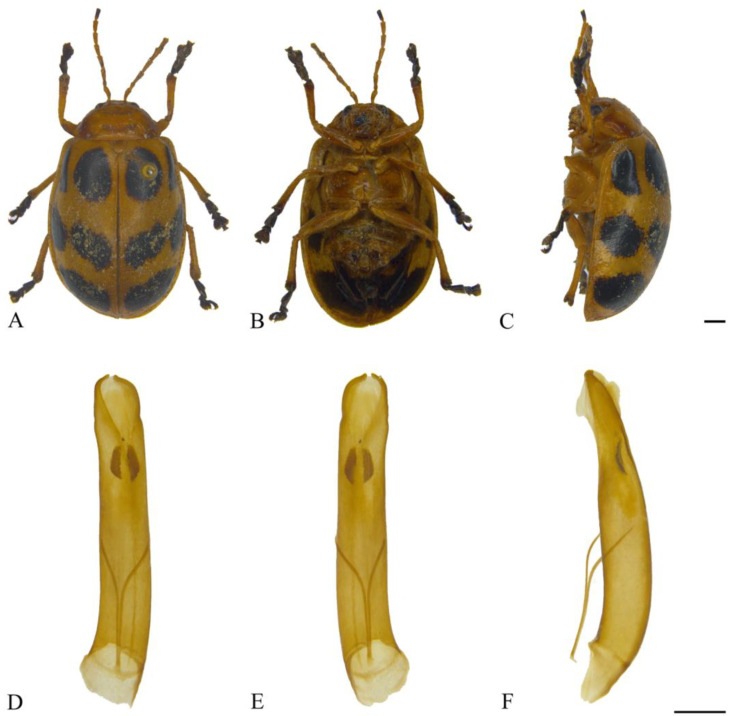
*Oides duporti* Laboissière. (**A**) Habitus (♀), dorsal view; (**B**) habitus (♀), ventral view; (**C**) habitus (♀), lateral view; (**D**) aedeagus, dorsal view; (**E**) aedeagus, ventral view; (**F**) aedeagus, lateral view. Scale bars: 1mm.

**Specimens examined.** China: 1♂1♀, Yunnan Prov., Funing Country, 1830 m, 9 May 1979, leg. Wang Dekou.

**Distribution.** China: Yunnan; Vietnam; Laos; Myanmar.

**Host plants. ***Illicium verum* (Hook. F.), *Schisandra chinensis* (Turczaninow).

**Remarks:** Lee & Beenen (2017) think *Oides duporti* Laboissière does not distribute in China, and the records from China should refer to *O. leucomelaena* Weise [1]. In this study, we found this species recorded in Yunnan, China.

#### 3.2.7. *Oides epipleuralis* Laboissière, 1929

*Oides epipleuralis* Laboissière, 1929: 254 [20]. Synonymized with *Oides laticlava* (Fairmaire, 1889) by Gressitt & Kimoto, 1963: 478 [19].

*Oides epipleuralis*: Lee & Beenen, 2017:48 [1]. Removed from synonymy of *Oides laticlava* (Fairmaire, 1889).

No specimen was examined.

**Distribution.** China: Taiwan.

#### 3.2.8. *Oides innocua* Gahan, 1891 (Figure 8A–C)

*Oides innocua* Gahan, 1891: 457 [22].

*Oides kanaraensis* Jacoby, 1904: 393 [27]. Synonymized by Maulik, 1936: 120 [28].

*Oides bengalensis* Maulik, 1936: 110 [28]. Synonymized by Lee & Beenen, 2017: 65 [1].

**Figure 8:**
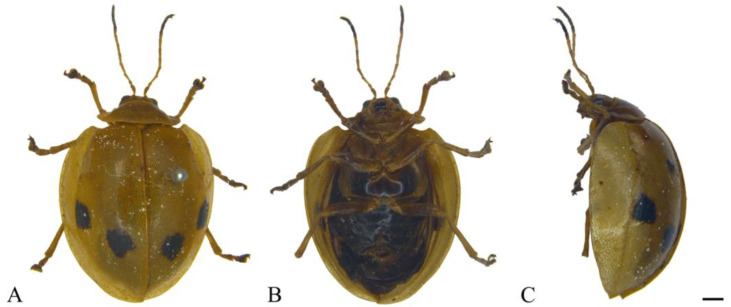
*Oides innocua* Gahan (♀). (**A**) Habitus, dorsal view; (**B**) habitus, ventral view; (**C**) habitus, lateral view. Scale bars: 1mm.

**Description. Male.** Length 11.5 mm, width 8.9 mm.

Body yellowish-brown. Antennae yellow, antennomeres VIII–XI black; Elytra yellowish-brown, each elytra with two black rounded spots; metasternum black; leg yellowish-brown; abdomen black. Antennae filiform, 1/3 the length of body; antennomere I dilated, II shortest, IV longest, III slightly shorter than IV; V–VII subequal in length, slightly shorter than III; VIII–X subequal in length, slightly shorter than VII; XI slightly longer than X, subequal to VII in length. Pronotum transverse, more than 2.0× wider than long; anterior margin concaved, front angles protrude anteriorly, back angles obtuse, disc smooth, without punctures. Scutellum tongue-like, smooth, without punctures. Elytra oval, disc with dense punctures; epipleurae slightly narrower than 1/2 the width of elytra.

**Specimens examined.** China: 1♀, Guangxi Prov., Napo Country, Defu, 100 m, 16 August 1998, leg. He Tongli.

**Distribution.** China: Guangxi (this study); India; Bangladesh.

**Remarks:** In this study, we only examined one female specimen collected from Napo, Guangxi, China. The characteristic that each elytra with one pair of black spots is consistent with *Oides innocua* Gahan, but the color of antennomeres VIII–XI and abdomen is black which is different from that of *O. innocua.* Therefore, the accuracy of this species still needs further confirmation.

#### 3.2.9. *Oides laticlava* (Fairmaire, 1889) (Figure 9A–F)

*Adorium laticlavum* Fairmaire, 1889: 74 [29]. Synonymized with *Oides maculata* (Oliver, 1807) by Kimoto, 1989: 38 [21].

*Oides laticlava*: Weise, 1924: 4 [15].

*Oides laticlavata* [sic!]: Laboissière, 1929: 252 [20].

*Oides laticlava*: Lee & Beenen, 2017: 68 [1]. Removed from synonymy of *Oides maculata* (Oliver, 1807)

**Figure 9:**
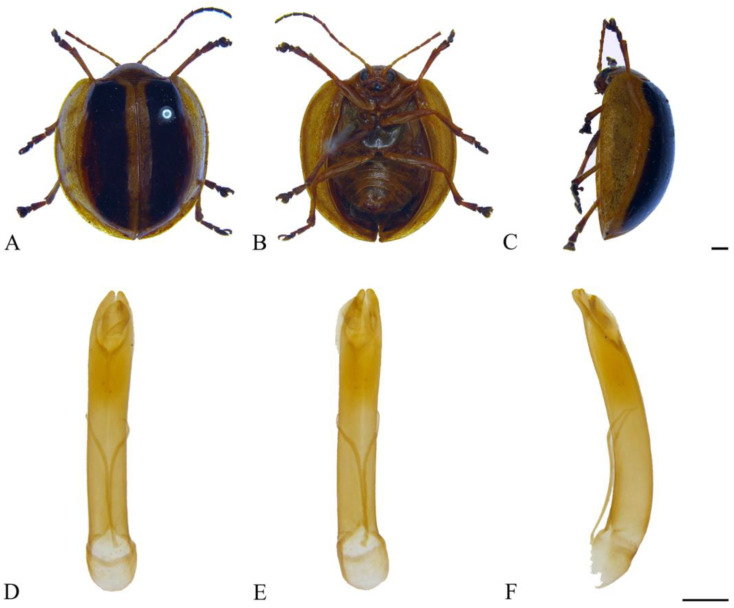
*Oides laticlava* (Fairmaire) (♂). (**A**) Habitus, dorsal view; (**B**) habitus, ventral view; (**C**) habitus, lateral view; (**D**) aedeagus, dorsal view; (**E**) aedeagus, ventral view; (**F**) aedeagus, lateral view. Scale bars: 1 mm.

**Specimens examined.** China: 1♂, Henan Prov., Mt. Jigong, June 1963, collector unknown; 1♂, Gansu Prov., Kang Country, Mt. Baiyun, 1250–1750 m, 12 July 1998, leg. Wang Shuyong; 1♂, Shaanxi Prov., Xunyang Country, 21 July 1960, collector unknown; 2♂, Shaanxi Prov., Ankang City, Qiaoting, 24 July 1981, leg. Huang Baoding; 1♂, Shaanxi Prov., Taibai Country, longwo, August 1980, leg. Zhang Zhixian; 1♂, Shaanxi Prov., Yang Country, Huayang Town, 27 June 2012, leg. Chen Ying; 1♂, Shaanxi Prov., Liuba Country, Miaotaizi, 1300 m, 27 July 1973, leg. Zhang Xuezhong; 1♀, Anhui Prov., Huoshan Country, Majia River, 900 m, 2 September 1978, leg. Wang Shuyong; 1♂, Zhejiang Prov., Mt. Tianmu, 14 August 1935, collector unknown; 1♂, Hubei Prov., Shennongjia, Jiuhu Town, 1800 m, 7 August 1987, leg. Han Yinheng; 1♀, Jiangxi Prov., Guling Town, 14 September 1934, collector unknown; 1♂, Hunan Prov., Mt. Heng, 22 August 1979, leg. Liu Jupeng; 1♀, Fujian Prov., Nanping City, Jianyang District, Huangkeng Town, Aotou Village, 800–950 m, 10 July 1960, leg. Zuo Yong; 1♀, Guangdong Prov., Lian Country, Zian Village, 28 June 1965, leg. Zhang Youwei; 1♀, Guangxi Prov., Xiuren Town, 7 October 1943; 1♀, Sichuan Prov., Nanjiang Country, 1400–1500 m, 12 August 1958, leg. Song Shimei; 1♂, Guizhou Prov., Guiding Country, 6 July 1930, collector unknown; 1♂, Guizhou Prov., Mt. Fanjing, Huguo Temple, 1350 m, 3 August 2001, leg. Song Qiongzhang.

**Distribution.** China: Henan, Shaanxi, Gansu, Anhui, Zhejiang, Hubei, Jiangxi, Hunan, Fujian, Guangdong, Hainan, Hongkong, Guangxi, Sichuan, Guizhou.

#### 3.2.10. *Oides leucomelaena* Weise, 1922 (Figure 10A–F)

*Oides leucomelaena* Weise, 1922: 58 [30]. Synonymized with *Oides duporti* Laboissière, 1919 by Kimoto, 1989: 36 [21].

*Oides leucomelaena* var. *subsinuata* Pic, 1928: 29 [31]. Synonymized by Kimoto, 1989: 36 [21].

*Oides leucomelaena* var. *disjuncta* Pic, 1928: 29 [31]. Synonymized by Kimoto, 1989: 36 [21].

*Oides leucomelaena*: Samoderzhenkov, 1992: 108 [32]. Removed from synonymy of *Oides duporti* Laboissière, 1919.

**Figure 10:**
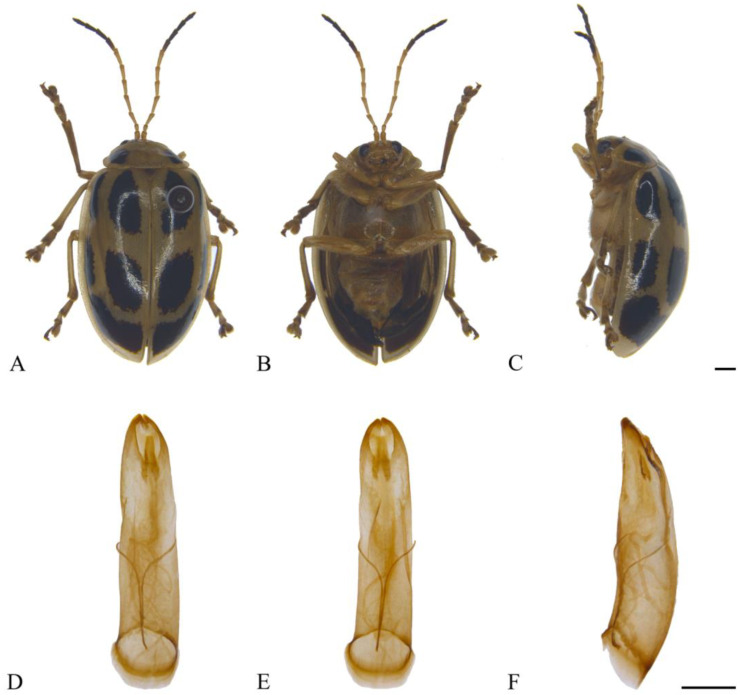
*Oides leucomelaena* Weise (♂). (**A**) Habitus, dorsal view; (**B**) habitus, ventral view; (**C**) habitus, lateral view; (**D**) aedeagus, dorsal view; (**E**) aedeagus, ventral view; (**F**) aedeagus, lateral view. Scale bars: 1 mm.

**Specimens examined.** China: 1♀, Zhejiang Prov., Longquan City, 22 August 1982, leg. Chen Qihu; 1♂, Fujian Prov., Chongan Country, Xing Village, Longdu, 580–640 m, 10 July 1960, leg. Ma Chenglin; 1♂, Guangdong Prov., Daqiling, 1954, collector unknown; 1♂, Hainan Prov., Baisha Country, Mt. Yanggeling, Nanmaola, 11 May 2009, leg. Dang Lihong; 1♂, Guangxi Prov., Napo Country, Defu, 24 May 1998, leg. Yang Xingke; 1♀, Sichuan Prov., Mt. Emei, Hongchunping, 2–3 August 2011, leg. Zheng Lihao; 1♀, Guizhou Prov., Daozhen Country, Dashahe nature reserve, Mopanshi, 1600–1700 m, 17–20 August 2004, leg. Song Qiongzhang; 2♂, Yunnan Prov., Pingbian Country, Mt. Dawei, 1900 m, 23 June 1956.

**Distribution.** China: Zhejiang, Anhui, Hubei, Fujian, Guangdong, Hainan, Guangxi, Sichuan, Guizhou, Yunnan; Vietnam; Laos.

#### 3.2.11. *Oides livida* (Fabricius, 1801) (Figure 11A–F)

*Adorium lividum* Fabricius, 1801: 410 [4]. 

*Oides livida* Weber, 1801: 53 [3]. Synonymized by Harold, 1876: 3555 [5].

*Oides analis* Schönherr, 1808: 231 [33]. Synonymized by Harold, 1876: 3555 [5].

*Adorium diardi* Guérin-Méneville, 1830: 148 [34]. Synonymized by Kimoto, 1989: 37 [21].

*Rhombopalpa pectoralis* Clark, 1865: 144 [13]. Synonymized by Vachon, 1980: 144 [35].

*Oides nigripes* Jacoby, 1891: 34 [36]. Synonymized with *Oides pectoralis* Clark, 1865 by Gahan, 1891: 457 [22].

*Oides pallidicornis* Jacoby, 1899: 284 [37]. Synonymized by Vachon, 1980: 15 [35].

*Oides livida*: Wilcox, 1971: 11 [38].

**Figure 11:**
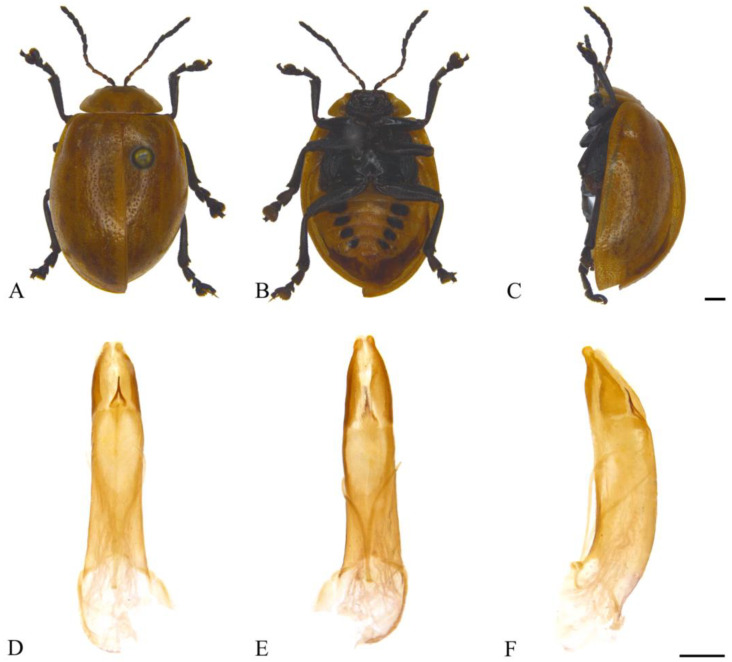
*Oides livida* (Fabricius) (♂). (**A**) Habitus, dorsal view; (**B**) habitus, ventral view; (**C**) habitus, lateral view; (**D**) aedeagus, dorsal view; (**E**) aedeagus, ventral view; (**F**) aedeagus, lateral view. Scale bars: 1 mm.

**Specimens examined.** China: 1♀, Guangxi Prov., Fangcheng, Fulong, 200 m, 25 May 1999, leg. Li Wenzhu; 1♂, Guangxi Prov., Jinxiu City, Mt. Shengtang, 900 m, 19 May 1999, leg. Han Hongxiang; 1♂, Yunnan Prov., Xishuangbanna, Menglun, 650 m, 19 May 1964, leg. Zhang Baolin.

**Distribution.** China: Fujian, Guangdong, Guangxi, Guizhou, Yunnan, Xizang; Vietnam; Laos; Thailand; India; Myanmar; Nepal; Buhtan; Malaysia; Singapore; Indonesia; Bangladesh.

#### 3.2.12. *Oides maculata* (Olivier, 1807) (Figure 12A–F)

*Adorium maculatum* Oliver, 1807: 611 [39].

*Adorium subhemisphaericum* Guérin-Méneville, 1830: 146 [34]. Synonymized by Maulik, 1936: 113 [28].

*Oides maculata:* Harold, 1876: 3555 [5].

**Figure 12:**
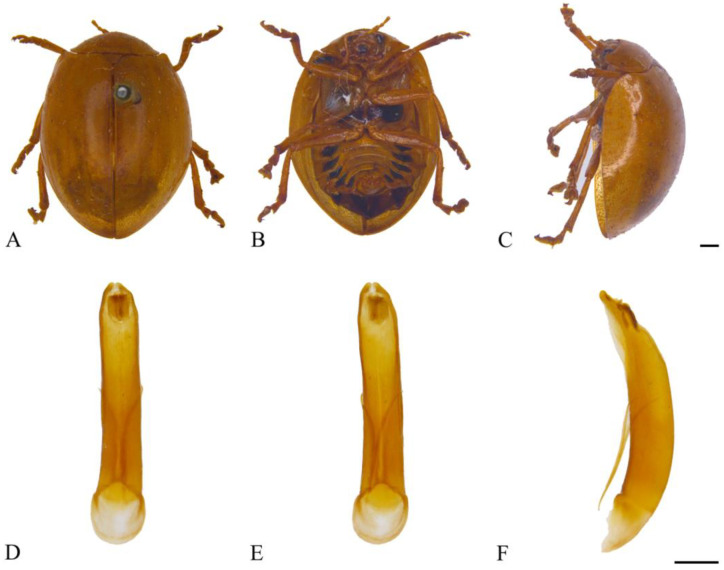
*Oides maculata* (Olivier) (♂). (**A**) Habitus, dorsal view; (**B**) habitus, ventral view; (**C**) habitus, lateral view; (**D**) aedeagus, dorsal view; (**E**) aedeagus, ventral view; (**F**) aedeagus, lateral view. Scale bars: 1 mm.

**Specimens examined.** China: 1♂, Guangxi Prov., Ningming Country, Longrui, 22 May 1984, leg. Meng Tian; 1♂, Xizang, Motuo Country, Dijing, 1000 m, 4 June 1983, leg. Lin Zai.

**Distribution.** China: Guangxi, Xizang; Vietnam; Laos; Thailand; Cambodia; India; Myanmar; Nepal; Indonesia; Bangladesh.

**Host plants. ***Vitis* sp., *Corylus* sp.

#### 3.2.13. *Oides multimaculata* Pic, 1928 (Figure 13A–F)

*Oides multimaculata* Pic, 1928: 29 [31].

**Specimens examined.** China: 1♂, Yunnan Prov., Xishuangbanna, Menghun, 1200–1400 m, 23 May 1958, leg. Zhang Yiran.

**Distribution.** China: Yunnan; Vietnam; Laos.

**Figure 13:**
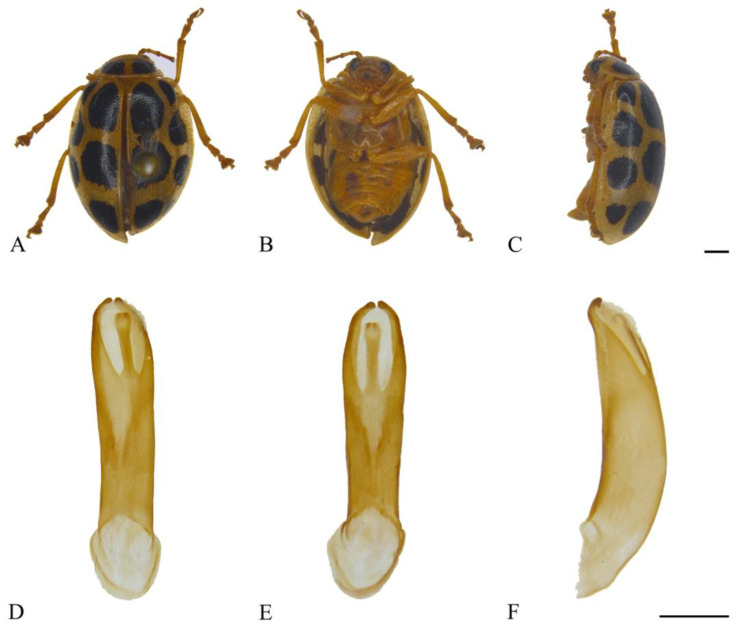
*Oides multimaculata* Pic (♂). (**A**) Habitus, dorsal view; (**B**) habitus, ventral view; (**C**) habitus, lateral view; (**D**) aedeagus, dorsal view; (**E**) aedeagus, ventral view; (**F**) aedeagus, lateral view. Scale bars: 1 mm.

#### 3.2.14. *Oides palleata* (Fabricius, 1781) (Figure 14A–F)

*Chrysomela bipunctata* Fabricius 1781: 127 [40] (nec Linnaeus, 1758).

*Adorium palleatum* Fabricius, 1801: 410 [4].

*Oides palleata:* Harold, 1876: 3556 [5].

*Oides indosinensis* Laboissière, 1927: 37 [24]. Synonymized with *Oides andrewesi* Jacoby, 1900 by Gressitt & Kimoto, 1963: 476 [19]. Synonymized by Lee & Beenen, 2017: 90 [1].

*Oides indosinensis* var. *Laboissièrei* Pic, 1927: 23 [41]. Synonymized with *Oides andrewesi* Jacoby, 1900 by Wilcox, 1971: 3 [38]. Synonymized by Lee & Beenen, 2017: 90 [1].

**Specimens examined.** China: 2♂1♀, Hainan Prov., Baisha Country, Shifu Village, 19°00′69″ N, 109°36′75″E, 392 m, 15 May 2009, leg. Huang Xinlei; 1♀, Hainan Prov., Baisha Country, Mt. Yingge, Hongxin Village, 19°06′89″ N, 109°52′39″ E, 600 m, 17 June 2008, leg. Shi Baoliang; 1♀, Guangxi Prov., Longrui, 1020 m, 20 May 1984, leg. Wang Shufang; 2♂, Guangxi Prov., Pingxiang City, 14 June 1976, leg. Zhang Baolin; 2♀, Sichuan Prov., Wenchuan Country, 3 June 1953, collector unknown; 1♀, Yunnan Prov., Xishuangbanna, Menglun, 650 m, 25 July 1959, leg. Pu Fuji; 1♂, Yunnan Prov., Xishuangbanna, Mengla, 620–650 m, 10 June 1959, leg. Zhang Yiran.

**Distribution.** China: Hainan, Guangxi, Sichuan, Guizhou, Yunnan; Vietnam; Laos; Thailand; Cambodia; India; Myanmar; Nepal; Indonesia; Bangladesh.

**Figure 14:**
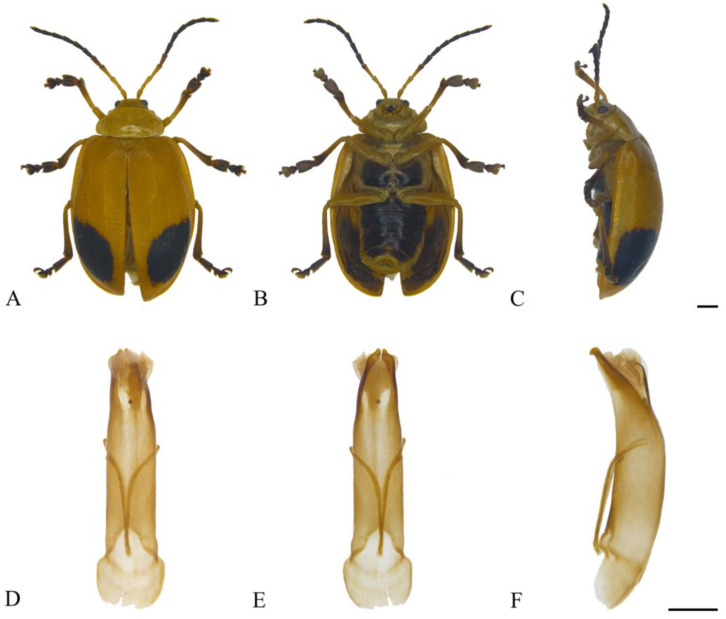
*Oides palleata* (Fabricius) (♂). (**A**) Habitus, dorsal view; (**B**) habitus, ventral view; (**C**) habitus, lateral view; (**D**) aedeagus, dorsal view; (**E**) aedeagus, ventral view; (**F**) aedeagus, lateral view. Scale bars: 1 mm.

#### 3.2.15. *Oides paraboreri* **sp. nov.** (Figure 15A–H)

**Description. Male.** Length 10.0–11.0 mm, width 8.0–9.2 mm. **Female.** Length 10.5–12.8 mm, width 8.5–10.0 mm.

Body yellow or yellowish-brown. Antennae yellow, antennomere IV apex and VII–XI black; metasternum yellowish-brown, few specimens dark brown; leg yellow or yellowish-brown; abdomen yellowish-brown, few specimens dark brown. Antennae filiform, slightly shorter than 1/2 the length of body; antennomere I dilated, II shortest, IV longest, III slightly shorter than IV; V–VII subequal in length, slightly shorter than IV; VIII–X subequal in length, slightly shorter than VII; XI slightly longer than X, subequal to VII in length. Pronotum transverse, more than 2.0× wider than long; anterior margin concaved, front angles protrude anteriorly, back angles obtuse, disc smooth, without punctures. Scutellum tongue-like, smooth, without punctures. Elytra oval, surface with dense punctures, diameter of puncture shorter than spacing between punctures; epipleurae 1/2 the width of elytra. Aedeagus slender, nearly parallel-sided; apex bifurcated, beak-like in lateral view. Female sternite VIII weakly sclerotized, fanshaped, apical region depressed medially with long setae, spiculum long. Gonocoxae reduced. Spermatheca strongly curved and hook-like; proximal end of basal semitransparent, distal end membranous and constricted; middle region narrowed from distal to proximal end; apex widely rounded.

**Holotype:** ♂, China, Sichuan Prov., Mt. Emei, 2 August 1992, leg. Yuan Decheng. **Paratypes:** China: 1♂1♀, Guangxi Prov., Nanning City, Mt. Daming, Lingying Villa, 1424 m, 21–23 July 2019, 23.47850° N, 108.43146° E, leg. Yan Changpeng; 2♂2♀, Sichuan Prov., Wan Country, Wang’erbao, 1300 m, 29 September 1994, leg. Song Shimei; 1♂, Sichuan Prov., Wan Country, Wang’erbao, 1300 m, 30 September 1994, leg. Song Shimei; 1♂, Sichuan Prov., Wan Country, Wang’erbao, 1300 m, 15 August 1993, leg. Song Shimei; 1♂, Sichuan Prov., Wan Country, Wang’erbao, 1200 m, 27 September 1994, leg. Yao Jian; 1♀, Sichuan Prov., Wan Country, Wang’erbao, 1300 m, 9 July 1994, leg. Huang Runzhi; 1♂, Sichuan Prov., Mt. Emei, Baoguo Temple, 500 m, 22 July 1974, leg. Zhou Yao and Yuan Feng (NWAFU).

**Diagnosis.** The new species is similar to *Oides boreri* Lee *et* Beenen, but antennomeres VI-XI black; pronotum without punctures; meso- and metasternum, abdomen yellowish-brown, few specimens dark brown; morphology of aedeagus apex is completely different from the latter.

**Etymology.** The specific name refers to this new species similarity to *Oides boreri* Lee et Beenen.

**Distribution.** China (Figure 2): Guangxi, Sichuan; Laos.

**Figure 15:**
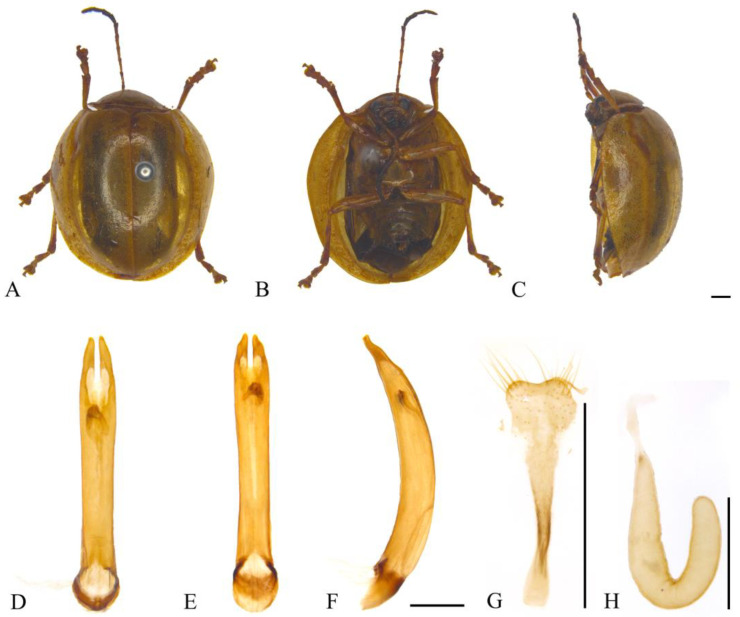
*Oides paraboreri ***sp. nov.** (**A**) Habitus, dorsal view; (**B**) habitus, ventral view; (**C**) habitus, lateral view; (**D**) aedeagus, dorsal view; (**E**) aedeagus, ventral view; (**F**) aedeagus, lateral view; (**G**) female sternite VIII; (**H**) spermatheca. Scale bars: 1 mm.

#### 3.2.16. *Oides parabowringii* **sp. nov.** (Figure 16A–I)

**Description. Male.** Length 12.0–13.5 mm, width 8.0–9.5 mm. **Female.** Length 13.1 mm, width 8.9 mm.

**Figure 16:**
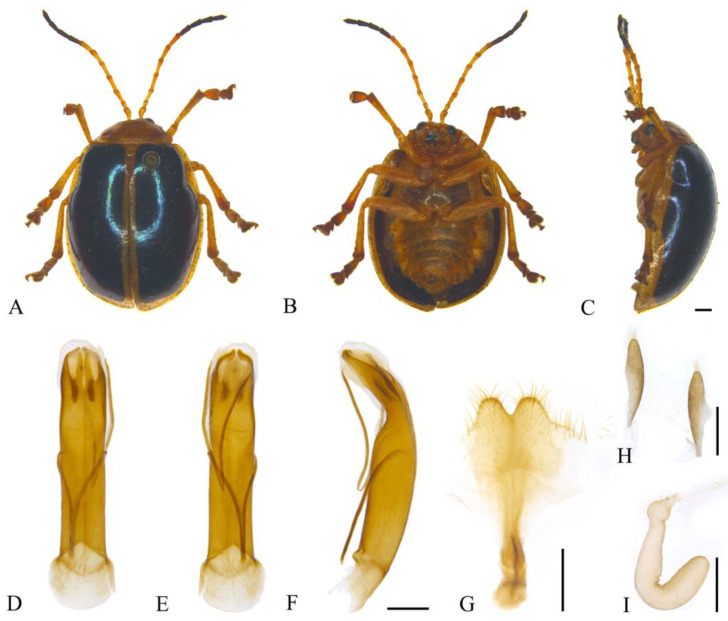
*Oides parabowringii ***sp. nov.** (**A**) Habitus, dorsal view; (**B**) habitus, ventral view; (**C**) habitus, lateral view; (**D**) aedeagus, dorsal view; (**E**) aedeagus, ventral view; (**F**) aedeagus, lateral view; (**G**) female sternite VIII; (**H**) gonocoxae; (**I**) spermatheca. Scale bars: 1 mm.

Body yellow. Antennomeres VIII–XI black; disc of pronotum dark brown; elytra metallic blue with mesal sutural edge, back and outer margins yellow; leg yellow, tarsus, and pretasus black; abdomen yellow. Antennae filiform, slightly shorter than 3/4 the length of body; antennomere I dilated, II shortest, IV longest, III slightly shorter than IV; V–VII subequal to III in length; VIII–XI subequal in length, slightly shorter than VII. Pronotum transverse, slightly less 2.5× wider than long; anterior margin concaved, front angles protrude anteriorly, back angles obtuse, disc with dense punctures. Scutellum tongue-like, smooth, without punctures. Elytra oval, disc with dense punctures, diameter of puncture distinctly shorter than spacing between punctures; epipleurae about 1/5 the width of elytra. Aedeagus nearly parallel-sided with apex bifurcated in dorsal view; terminal of apical processes separated in ventral view; aedeagus apex strongly constricts ventrally at apical 1/3 in lateral view. Female sternite VIII sclerotized, fanshaped, apex projecting anteriorly, strongly depressed medially, with long setae, spiculum moderately long. Gonocoxae separated and longitudinal, slightly fusiform, apex with setae, basal narrower than apex. Spermatheca hook-like, weakly sclerotized, distal end of basal region strongly dilated; proximal end of middle slightly narrowed; apical region wider than middle, apex slightly narrowed and widely rounded.

**Holotype:** ♂, China, Sichuan Prov., Mt. Emei, Jiulaodong, 1800–1900 m, 14 August 1957, leg. Zhu Fuxing. **Paratypes:** China: 1♂, Jiangxi Prov., Longnan City, Mt. Jiulian, 18 June 1975, leg. Zhang Youwei; 1♂, Jiangxi Prov., Mt. Jiulian, Huangniushi, 17 June 1975, leg. Zhang Youwei; 1♀, Fujian Prov., Chong’an Country, Tongmuguan, Guanping, 800–1000 m, 21 July 1960, leg. Pu Fuji; 1♂, Guizhou Prov., Chishui Country, Jinsha Village, 500 m, 23 September 2000, IOZ & Guizhou Univ. Joint Expedition, leg. Liang Hongbin.

**Diagnosis.** The new species is similar to *Oides bowringii* (Baly), but mesal sutural edge, back, and outer margins of elytra are yellow; aedeagus is broader, terminal of apical processes separated in ventral view.

**Etymology.** The specific name refers to this new species similarity to *Oides bowringii* (Baly).

**Distribution.** China (Figure 2): Jiangxi, Fujian, Guizhou.

#### 3.2.17. *Oides parathibettana* **sp. nov.** (Figure 17A–F)

**Description. Male.** Length 11.8–12.0 mm, width 8.5–8.9 mm.

**Figure 17:**
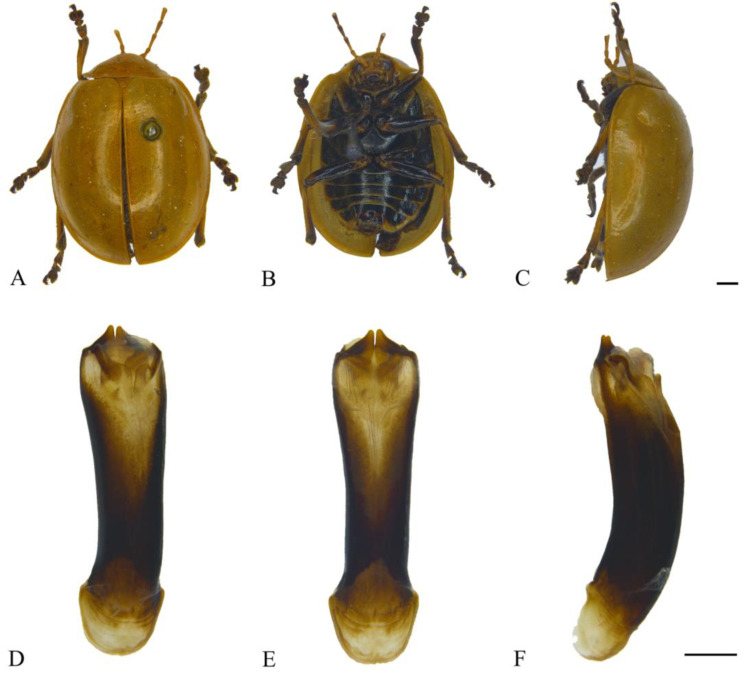
*Oides parathibettana* **sp. nov.** (♂). (**A**) Habitus, dorsal view; (**B**) habitus, ventral view; (**C**) habitus, lateral view; (**D**) aedeagus, dorsal view; (**E**) aedeagus, ventral view; (**F**) aedeagus, lateral view. Scale bars: 1 mm.

Body yellow or yellowish-brown. Antennomeres VIII–XI black; leg yellow or yellowish-brown, femur and ventral parts of tibia and tarsus black; abdomen black. Antennae filiform, 2/3 the length of body; antennomere I dilated, II shortest, III longest, IV distinctly shorter than III; V–VII subequal in length, slightly shorter than IV; VIII–X subequal in length, slightly shorter than VII; XI slightly longer than X, subequal to VII in length. Pronotum transverse, more than 2.0× wider than long; anterior margin concaved, front angles protrude anteriorly, back angles obtuse, disc smooth, without punctures. Scutellum tongue-like, smooth, without punctures. Elytra oval, disc with dense punctures, diameter of puncture nearly equal to spacing between punctures in length; epipleurae narrower than 1/3 the width of elytra. Aedeagus short and thick, evenly widening from basal 1/2 to apex, slightly wider than base; apex bifurcated, terminal of apical processes acute; aedeagus constricts dorsally at apical 1/3 in lateral view.

**Holotype:** ♂, China, Gansu Prov., Hui Country, Yuguan Village, 23 May 1981, collector unknown. **Paratypes:** China: 1♂, Shaanxi Prov., Liping, 1000–1200 m, 19 June 1958, leg. Song Shimei; 2♂, Sichuan Prov., Ya’an, date and collector unknown.

**Diagnosis.** The new species is similar to *Oides thibettana* Jacoby, but antennomeres VIII–XI are black; femur and ventral parts of tibia and tarsus are black; pronotum has no punctures; aedeagus is short and thick, adeagus apex is slightly rectangular in dorsal and ventral view.

**Etymology.** The specific name refers to this new species similarity to *Oides thibettana* Jacoby.

**Distribution.** China (Figure 2): Gansu, Shaanxi, Sichuan.

#### 3.2.18. *Oides scutellata* (Hope, 1831) (Figure 18A–C)

*Adorium scutellatum* Hope, 1831: 28 [42].

*Oides scutellata*, Harold, 1876: 3556 [5].

*Oides gyironga* Chen *et* Jiang, 1981: 468 [43]. Synonymized by Lee & Beenen, 2017: 98 [1].

**Specimens examined.** China: 1♂ (Holotype of *Oides gyironga* Chen *et* Jiang, abdomen lost), Xizang, Jilong Country, 2400 m, 22 July 1975, leg. Huang Fusheng.

**Distribution.** China: Xizang; Bhutan; India; Nepal; Pakistan.

**Figure 18:**
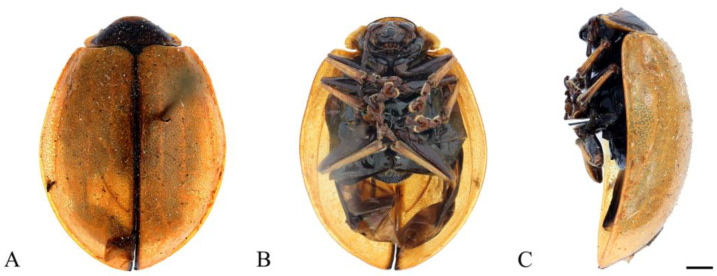
*Oides scutellata* (Hope) (♂). (**A**) Habitus, dorsal view; (**B**) habitus, ventral view; (**C**) habitus, lateral view. Scale bars: 1 mm.

#### 3.2.19. *Oides semipunctata* Duvivier, 1884 (Figure 19A–F)

*Oides semipunctata* Duvivier, 1884: 133 [44].

*Oides quadrimaculata* Jacoby, 1900: 126 [45]. Synonymized by Maulik, 1936: 116 [28].

**Specimens examined.** China: 1♂1♀, Yunnan Prov., Ruili City, 870 m, 13 June 1956, leg. Huang Tianrong.

**Distribution.** China: Yunnan; Vietnam; Laos; India; Myanmar; Nepal; Bangladesh.

**Figure 19:**
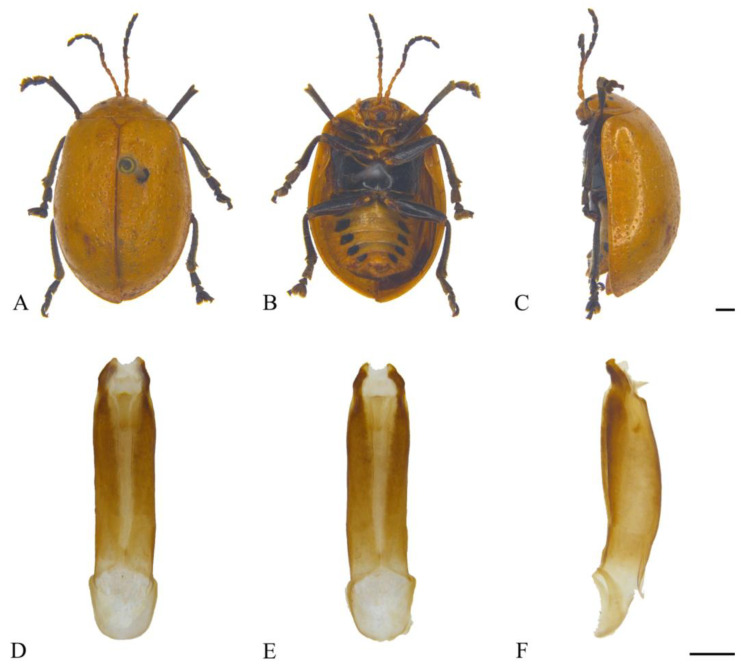
*Oides semipunctata* Duvivier (♂). (**A**) Habitus, dorsal view; (**B**) habitus, ventral view; (**C**) habitus, lateral view; (**D**) aedeagus, dorsal view; (**E**) aedeagus, ventral view; (**F**) aedeagus, lateral view. Scale bars: 1 mm.

#### 3.2.20. *Oides shimenensis* **sp. nov.** (Figure 20A–F)

**Description. Male.** Length 11.5 mm, width 8.5 mm.

Body yellow. Antennomeres IX–XI, metapleura and metasternum black; leg yellow, ventral part of tarsus dark brown; abdomen black, each ventrite with one pair of black longitudinal spots. Antennae filiform, slightly longer than 1/2 the length of body; antennomere I dilated, II shortest, III longest, IV slightly shorter than III; V–VII subequal in length, slightly shorter than IV; VIII–XI subequal in length, slightly shorter than VII. Pronotum transverse, slightly less 2× wider than long; anterior margin concaved, front angles protrude anteriorly, back angles obtuse, disc with dense punctures. Scutellum tongue-like, smooth without punctures. Elytra oval, disc with dense punctures, diameter of puncture distinctly shorter than spacing between punctures; epipleurae about 1/3 the width of elytra. Aedeagus evenly widens from apical 1/4 to 1/7, narrowing from apical 1/7 to apex, apex bifurcated; dorsal and ventral everted endophallus of aedeagus developed in lateral view.

**Holotype:** ♂, China, Hunan Prov., Shimen Country, Hupingshan Town, Sufu Village, 804 m, 15 October 2014, 30.0384° N, 110.8827° E, leg. Liang Hongbin. 

**Diagnosis.** The new species is similar to *Oides tarsata* (Baly), but antennomere III is the longest; IV is shorter than III; aedeagus constricts at apical 1/4 in dorsal view, apical processes longer, and terminal sharp; apex is not expanded in lateral view.

**Etymology.** The specific name is named after its type locality “Shimen”.

**Distribution.** China (Figure 2): Hunan.

**Figure 20:**
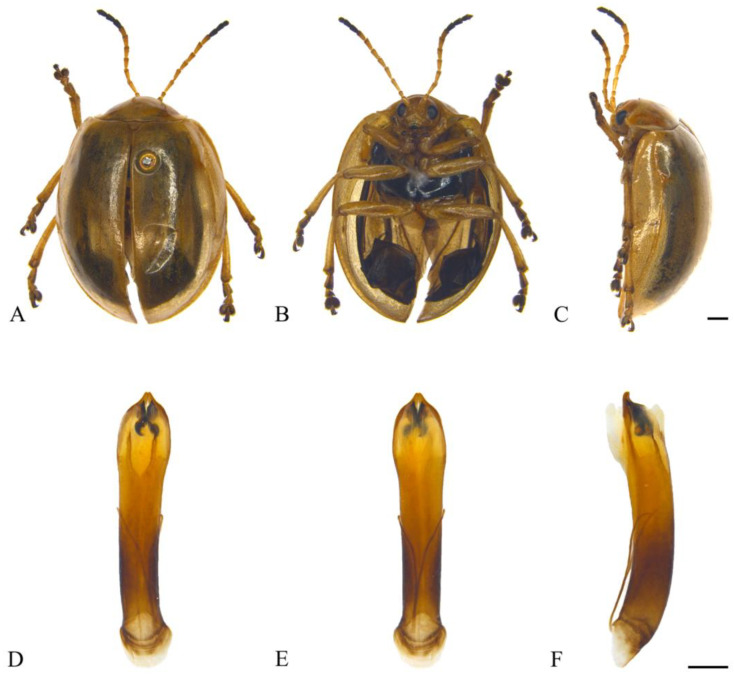
*Oides shimenensis* **sp. nov.** (♂). (**A**) Habitus, dorsal view; (**B**) habitus, ventral view; (**C**) habitus, lateral view; (**D**) aedeagus, dorsal view; (**E**) aedeagus, ventral view; (**F**) aedeagus, lateral view. Scale bars: 1 mm.

#### 3.2.21. *Oides tarsata* (Baly, 1865) (Figure 21A–F)

*Adorium tarsatum* Baly, 1865: 435 [46].

*Adorium sordidum* Baly, 1865: 435 [46]. Synonymized by Gressitt & Kimoto, 1963: 479 [19].

*Oides tarsata*: Harold, 1876: 3556 [5].

*Oides indica* Baly, 1879: 443 [47]. Synonymized by Lee & Beenen, 2017: 104 [1].

*Oides chinensis* Weise, 1922: 57 [30]. Synonymized by Lee & Beenen, 2017: 104 [1].

**Specimens examined.** China: 1♂, Henan Prov., Shangcheng Country, Suxianshi Town, Xihe scenic zone, 420.73 m, 5 October 2021, 31.72967° N, 115.54433° E, village, shrub, leg. Liang Hongbin and Chen Jun; 2♂, Henan Prov., Lushan Country, 28 May 1957, collector unknown; 1♂, Shaanxi Prov., Ningxi Forestry Bureau Puhe Forest Farm, mulberry, May 1981, collector unknown; 1♂, Shaanxi Prov., Mt. Hua, 1000 m, 10 August 1972, leg. Wang Shuyong; 1♂, Shaanxi Prov., Zhen’an Country, 31 May 1975, collector unknown (NWAFU); 2♂, Anhui Prov., Jinzhai Country, Nanxi Town, Geshan Village, 231.13 m, 4 May 2021, 31.55304° N, 115.57138° E, village, shrub, leg. Zhao Kaidong and Zhu Xichao; 1♂, Anhui Prov., Jinzhai Country, Tangjiahui Town, near Jintaoyuan Business Hotel, 190.92 m, 1 May 2021, 31.60996° N, 115.58233° E, pond, woods, leg. Zhao Kaidong and Zhu Xichao; 1♂, Anhui Prov., Susong Country, Zhifeng Town, Baiyazhai scenic zone, 155.17 m, 21 May 2021, 30.36677° N, 116.14921° E, roadside, shrub, leg. Zhu Pingzhou; 1♂, Zhejiang Prov., Hangzhou City, 18 May 1923, collector unknown; 1♂, Hubei Prov., Shennongjia, 900 m, pine and cypress, 7 June 1981, leg. Han Yinheng; 1♂, Hubei Prov., date and collector unknown; 1♂, Hubei Prov., Lichuan City, Mt. Xingdou, Sanxianchang Forest Farm, 1164 m, 19 May 2017, 30.0486° N, 109.1315° E, leg. Zhao Kaidong; 1♂, Fujian Prov., Hua’an Country, Wenhua Village, 10 July 1981, collector unknown; 1♂, Hainan Prov., Mt. Diaoluo, 1000 m, 23 April 1980, leg. Wang Shuyong; 1♂, Hainan Prov., Tianchi, 10 April 1980, leg. Wang Shuyong; 1♂, Guangxi Prov., Longsheng Country, Mt. Tianping, 740 m, 5 June 1963, leg. Wang Shuyong; 1♂, Guangxi Prov., Longsheng Country, Mt. Tianping, 740 m, 5 June 1963, leg. Wang Chunguang; 1♂, Guangxi Prov., Longzhou, Mt. Daqing, 360 m, 18 April 1963, leg. Shi Yongshan; 1♂, Guangxi Prov., Longzhou, Mt. Daqing, 16 June 1976, leg. Zhang Baolin; 1♂, Guangxi Prov., Jinxiu Country, Mt. Shengtang, 19 May 1999, leg. Xiao Hui; 1♂, Guizhou Prov., Leishan Country, Mt. Leigong, 1600 m, 30 June 1988, leg. Wang Shuyong.

**Distribution.** China: Hebei, Shandong, Henan, Shaanxi, Gansu, Anhui, Zhejiang, Hubei, Jiangxi, Hunan, Fujian, Guangdong, Hainan, Guangxi, Sichuan, Guizhou; Vietnam.

**Host plants. ***Vitis* spp., *Cayratia* spp.

**Figure 21:**
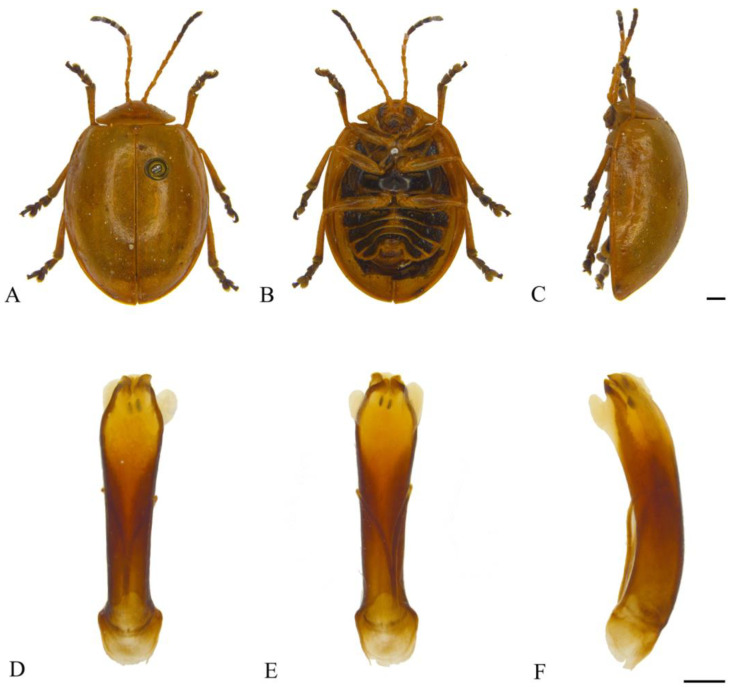
*Oides tarsata* (Baly, 1865) (♂). (**A**) Habitus, dorsal view; (**B**) habitus, ventral view; (**C**) habitus, lateral view; (**D**) aedeagus, dorsal view; (**E**) aedeagus, ventral view; (**F**) aedeagus, lateral view. Scale bars: 1 mm.

#### 3.2.22. *Oides thibettana* Jacoby, 1900

*Oides thibettana* Jacoby, 1900: 128 [45].

*Oides thibetana* [sic!]: Weise, 1924: 6 [15].

*Oides tibetana* [sic!]: Kimoto, 1989: 39 [21] (as synonym of *Oides tarsata* Baly, 1865*,* misidentification).

*Oides thibettana*: Lee & Beenen, 2017: 106 [1]. Removed from synonymy of *Oides tarsata* Baly, 1865.

No specimen was examined.

**Distribution.** China: Sichuan, Xizang; Myanmar.

#### 3.2.23. *Oides tibiella* Wilcox, 1971 (Figure 22A–F)

*Oides tibialis* Laboissière, 1927: 40 [24] (nec Duvivier, 1884).

*Oides tibiella* Wilcox, 1971: 17 [38] (new replacement name of *Oides tibialis* Laboissière, 1927).

*Oides tibiella*: Kimoto, 1989: 39 [21] (as synonym of *Oides tarsata* Baly, 1865, misidentification).

*Oides tibiella*: Lee & Beenen, 2017: 108 [1]. Removed from synonymy of *Oides tarsata* Baly, 1865.

**Specimens examined.** China: 1♂, Sichuan Prov., Mt. Emei, Jiulaodong, 27 August 1957, leg. Wang Zongyuan; 1♂, Yunnan Prov., Xishuangbanna, Mengla, 620–650 m, 29 May 1959, leg. Li Suofu; 1♂, Yunnan Prov., Xishuangbanna, Menghun, 1200–1400 m, 9 May 1958, leg. Hong Zhunpei.

**Distribution.** China: Sichuan, Yunnan; Vietnam; Laos; Thailand.

**Figure 22:**
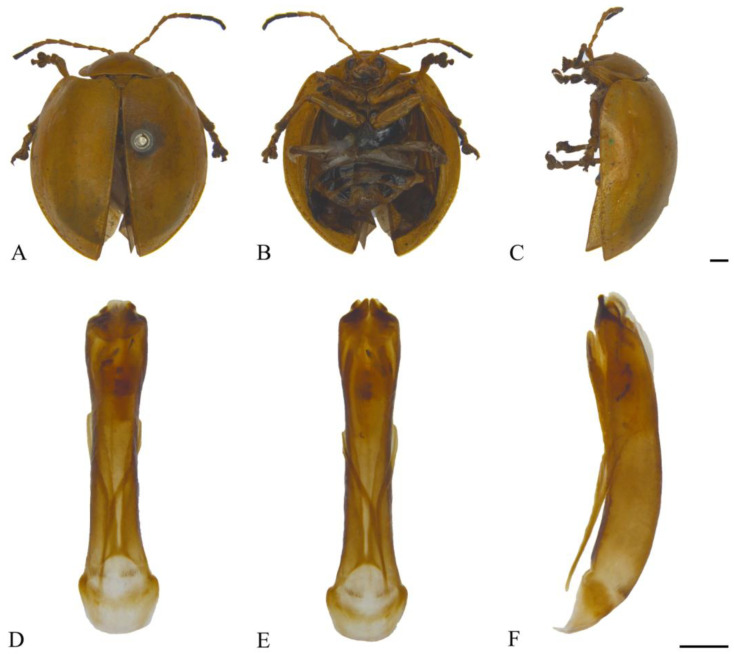
*Oides tibiella* Wilcox (♂). (**A**) Habitus, dorsal view; (**B**) habitus, ventral view; (**C**) habitus, lateral view; (**D**) aedeagus, dorsal view; (**E**) aedeagus, ventral view; (**F**) aedeagus, lateral view. Scale bars: 1 mm.

#### 3.2.24. *Oides ustulaticia* Laboissière, 1927 (Figure 23A–F)

*Oides ustulaticia* Laboissière, 1927: 41 [24].

**Specimen examined.** 2♂, Yunnan Prov., Pu’er City, 1080 m, 9 May 1956, leg. Zhou Benshou; 1♂, Yunnan Prov., Pu’er City, 1500 m, 12 May 1957, leg. Liu Dahua; 1♂1♀, Yunnan Prov., Lunan Country, Shilin, 16 May 1981, leg. Zhang Xuezhong; 1♂, Yunnan Prov., Weixi Country, Baijixun Village, 1780 m, 10 July 1981, grape, leg. Wang Shuyong.

**Distribution.** China: Guizhou, Yunnan.

**Host plant.** *Vitis vinifera* Linnaeus.

**Figure 23:**
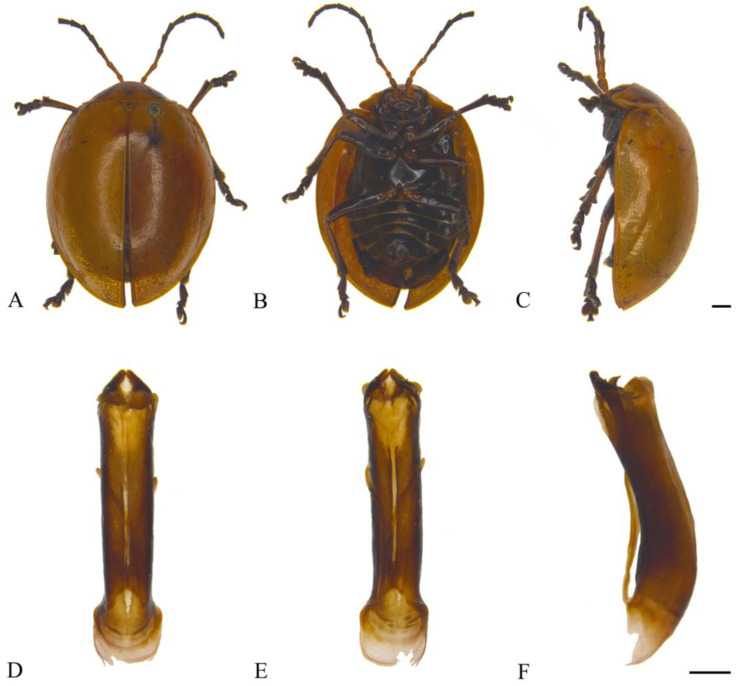
*Oides ustulaticia* Laboissière (♂). (**A**) Habitus, dorsal view; (**B**) habitus, ventral view; (**C**) habitus, lateral view; (**D**) aedeagus, dorsal view; (**E**) aedeagus, ventral view; (**F**) aedeagus, lateral view. Scale bars: 1 mm.

#### 3.2.25. *Oides yunnanensis* **sp. nov.** (Figure 24A–F)

**Description. Male.** Length 11.9 mm, width 8.0 mm.

Body yellow. Antennomeres IX–XI, mesopleura and mesosternum black; leg yellow, apex of tibia and ventral part of tarsus dark brown; abdomen yellow, each ventrite with one pair of black spots. Antennae filiform, 2/3 the length of body; antennomere I dilated, II shortest, III longest, IV–XI subequal in length, distinctly shorter than III. Pronotum transverse, more than 2.0× wider than long; anterior margin concaved, front angles protrude anteriorly, back angles obtuse, disc smooth, without punctures. Scutellum tongue-like, smooth without punctures. Elytra oval, disc with dense punctures, diameter of puncture longer than spacing between punctures; epipleurae narrower than 1/3 the width of elytra. Aedeagus slender, nearly parallel-sided; aedeagus apex with lobate processes in lateral view; terminal bent dorsally in lateral view.

**Holotype:** ♂, CHINA: Yunnan Prov., Chouzhai, 850 m, 10 May 1979, leg. Longling group.

**Diagnosis.** The new species is similar to *Oides tibiella* Wilcox, but antennomeres IX–XI are black; mesosternum and abdomen are yellowish-brown; each abdominal segment has one pair of black spots; aedeagus is slender, the shape of terminal processes is different.

**Etymology.** The specific name is named after its type locality “Yunnan”.

**Distribution.** China (Figure 2): Yunnan.

**Figure 24:**
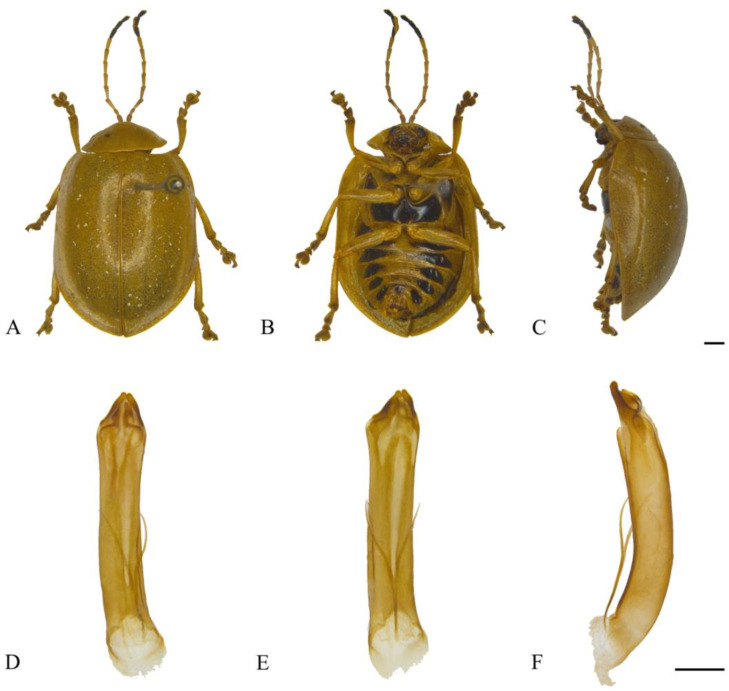
*Oides yunnanensis ***sp. nov.** (♂). (**A**) Habitus, dorsal view; (**B**) habitus, ventral view; (**C**) habitus, lateral view; (**D**) aedeagus, dorsal view; (**E**) aedeagus, ventral view; (**F**) aedeagus, lateral view. Scale bars: 1 mm.

### 3.3. Key to the Known Species of the Genus Oides from China

1. Elytra yellow············································································································· ········· ···2
  Elytra blue or with black spots···························································································16
2. Ventral side of body yellow························································*O. epipleuralis* Laboissiere
  Abdomen black or yellowish-brown with black spots······················································3
3. Leg entirely or partially black·······························································································4
  Leg yellow or tibia apex slightly black, tarsus black or blackish-brown·························9
4. Leg entirely black···················································································································5
  Leg partially black··················································································································7
5. Antennomeres 4–11 black; abdomen entirely black (Figure 23B)································································································*O. ustulaticia* Laboissiere
  Antennomeres 5–11 black; abdomen yellowish-brown, each segment with one pair of black spots··························································································································6
6. Head yellow, lateral front margin of pronotum with one pair of small black spots (Figure 19A,C)·············································································*O. semipunctata* Duvivier
  Head black (Figure 11B), pronotum without black spots (Figure 11A)··········································································································*O. livida* (Fabricius)
7. Antennae entirely black; pronotum with black spots; femur and tarsus black, tibia yellowish-brown (Figure 18A–C)························································*O. scutellata* (Hope)
  Antennae partially black; pronotum without black spots················································8
8. Antennomeres 9–11 black; tibia yellowish-brown; abdomen yellowish-brown, each segment with one pair of black spots (Figure 22B)·································*O. tibiella* Wilcox
  Antennomeres 8–11 black; tibia black ventrally, yellow dorsally; abdomen black (Figure 17B)···························································································*O. parathibettana* **sp. nov.**
9. Meso- and metathorax black ventrally···············································*O. thibettana* Jacoby
  Mesosternum yellow or yellowish-brown, metasternum black····································10
10. Antennomeres 6–11 black; metapleura yellowish-brown, metasternum black (Figure 12B)·······································································································*O. maculata* (Olivier)
  Antennomeres 7–11, 8–11 or 9–11 black; metapleura and metasternum yellowish-brown·····································································································································11
11. Abdomen entirely black ventrally·····················································································12
  Abdomen yellowish-brown ventrally, each segment with one pair of black spots·····13
12. Antennomeres 7–11 black (Figure 15B)···········································*O. paraboreri* **sp. nov.**
  Antennomeres 8–11 black (Figure 1B)················································*O. angusta* **sp. nov.**
13. Antennomeres 8–11 black, 3 equal to 4 (Figure 21B)···························*O. tarsata* (Baly)
  Antennomeres 9–11 black, 3 not equal to 4·····································································14
14. Pronotum without punctures (Figure 24A)································*O. yunnanensis* **sp. nov.**
  Pronotum with punctures···································································································15
15. Spacing between punctures on elytra longer than diameter of punctures (Figure 20A)···································································································*O. shimenensis* **sp. nov.**
  Spacing between punctures on elytra shorter than diameter of punctures (Figure 5A)··································································································*O. cystoprocessa* **sp. nov.**
16. Elytra black-blue··················································································································17
  Elytra with black spots or black longitudinal stripes·····················································18
17. Middle suture and disc of elytra black-blue (Figure 3A)···················*O. bowringii* (Baly)
  Disc of elytra black-blue and middle suture yellowish-brown (Figure 16A)·······························································································*O. parabowringii* **sp. nov.**
18. Elytra yellow, each elytra with a wide black longitudinal stripe in themiddle (Figure 9A)······································································································*O. laticlava* (Fairmaire)
  Elytra with black spots, not black longitudinal stripes····················································19
19. Each elytra with one large black spot behind the middle (Figure 14A)······································································································*O. palleata* (Fabricius)
  Each elytra with more than two black spots··································································20
20. Pronotum without black spots···························································································21
  Pronotum with black spots································································································23
21. Each elytra with two black spots behind the middle (Figure 8A)·······*O. innocua* Gahan
  Each elytra with five black spots························································································22
22. Antennomeres 9–11 black; leg yellow; each abdominal segment with one pair of black spots (Figure 6B)····································································*O. decempunctata* (Billberg)
  Antennomeres 8–11 black; tarsus black; abdomen yellowish-brown without spots (Figure 7B)···························································································*O. duporti* Laboissiere
23. Pronotum with four black spots, each elytra with five black spots (Figure 10A)·································································································*O. leucomelaena* Weise
  Pronotum with two black spots, elytra without five black spots·····························24
24. Each elytra with six black spots; meso- and metathorax black ventrally, abdomen yellowish-brown, each segment with one pair of black spots (Figure 4A,B)································································································*O. coccinelloides* Gahan
  Each elytra with seven black spots; body yellowish-brown ventrally without spots (Figure 13A,B)·····················································································*O. multimaculata* Pic

## 4. Discussion

The *Oides tarsata* species group is characterized by the yellow elytra, the epipleurae located 7/10 to 3/4 of the distance between suture and lateral margin, and elongated antennae with antennomeres VIII–X being more than 2.0× longer than wide, and *O. geiseri* Lee *et* Beenen, *O. tarsata* (Baly), *O. thibettana* Jacoby, *O. tibiella* Wilcox, and *O. ustulaticia* Laboissière are placed under this group [1]. Species of *Oides tarsata* species group can not be distinguished by external morphological characteristics and can only be identified via morphology of external genitalia [1]. In this study, five new species, including *O. yunnanensis* **sp. nov.**, *O. angusta* **sp. nov.**, *O. parathibettana* **sp. nov.**, *O. cystoprocessa* **sp. nov.**, and *O. shimenensis* **sp. nov.**, are put under this group. The type specimens of the new species described above are all identified from those previously classified as *O. tarsata* or *O. ustulaticia*. We believe that more cryptic species of *Oides* will be discovered in China.

In addition, Lee & Beenen (2017) doubted the distribution of *Oides flava* (Olivier) in China because no specimens were examined [1]. In our study, since any specimens of this species were found, we conclude that *O. flava* (Olivier) might be not distributed in China.

At present, 170 species of *Oides* are known in the world, and they are distributed in East Palaearctic, Indomalayan, Afrotropical, and Australian regions, with the most abundant species in the Australian region, followed by the Indomalayan region. Lee & Beenen (2017) systematically studied the species of Palaearctic and Oriental regions [1]. Berti (1993) described species in some areas of Africa [48]. Vachon (1977, 1980a, 1980b) revised the species of *Oides* in New Guinea and Southeast Asia, especially restudied the types of related species published by Fabricius in the genus *Adorium*, as well as types of New Guinean species mainly deposited in the Museum of Natural History, Paris (MNHN) and the Natural History Museum, London, UK (BMNH); a total of 22 species were studied, 16 new synonyms were identified, and 4 new species were published [35,49,50]. This study laid a good foundation for research on *Oides* in Australia. Except for the research mentioned above, no systematic studies and summaries of this genus have been conducted in the African and Australian regions. At present, we lack a comprehensive understanding of the species of this genus in the world, and a systematic summary is urgently needed.

The dorsal side of many species of genus *Oides* is yellow, such as the *Oides tarsata* species group, which can not be identified by external morphology, but only by differences in the male external genitalia. However, the lack of clear indicators on whether this difference is intraspecific or interspecific makes it difficult to apply. At the same time, the body color of some species varies greatly, and an effective method for how to define the range of variation within species has not been found. This will inevitably lead to the emergence of a large number of synonyms or cryptic species.

Because of the lack of comprehensive understanding of the species in the world, there must be obvious limitations or shortcomings in the understanding of the species group. The existing morphological data used for the classification of *Oides* groups are very imperfect and need to be reconstructed.

The study of types is the basic guarantee for the revision of species. The genus *Oides* has many problems concerning classification, such as some species have types from different species, types of some species were found not to belong to genus *Oides* at all, and even some synonymous types were found to be a different species, etc. Therefore, a systematic research on types of known species is necessary. Since some types of *Oides* have been lost or destroyed due to war, poor preservation, and other reasons, this brings some difficulties to the research work; the re-designation of new types has become a prominent problem [1].

In order to systematically revise and clarify phylogenetic relationship of *Oides*, it is necessary to re-establish the morphological data. At the same time, for species with similar morphologies or those difficult to distinguish by external morphology, the method of molecular systematics should be combined to solve the problem. In addition, we should pay attention to the species diversity and population structure of specific communities, and also clarify the distribution patterns and faunistic characteristics.

## Data Availability

The data presented in this study are available in this article.

## References

[1] Lee C.F., Beenen R. (2017). Revision of the Palaearetic and Oriental species of the genus *Oides* Weber, 1801 (Coleoptera: Chrysomelidae: Galerucinae). Zootaxa.

[2] Yang X.K., Ge S.Q., Nie R.E., Ruan Y.Y., Li W.Z. (2015). Chinese Leaf Beetles.

[3] Weber F. (1801). Observationes Entomologicae, Continets Novorum Quae Condidit Generum Characters, et Nuper Detectarum Specierum Descriptiones.

[4] Fabricius J.C. (1801). Systema Eleutheratorum Secundum Ordines, Genera, Species Adiectis Synonymis, Locis, Observationibus, Descriptionibus. Tomus I.

[5] van Harold E., Gemminger M., Harold B. (1876). Vol. XII: Chrysomelidae (Pars II), Languriidae, Erotylidae, Endomychidae, Coccinellidae, Corylophidae, Platypsyllidae. Catalogus Coleopterorum Hucusque Descriptorum Synonymicus et Systematicus.

[6] Billberg G.J. (1820). Enumeratio Insectorum in Museo Gust JohBillberg.

[7] Barber H.S. (1947). Diabrotiea and two new genera (Coleoptera, Chrysomelidae). Proc. Entomol. Soc. Wash..

[8] Chevrolat L.A.A., Dejean P.F.M.A. (1836). New taxa. Catalogue des Coléoptères de la Collection de le M. le Comte Dejean.

[9] Beenen R., Löbl I., Smetana A. (2010). Galerucinae. Catalogue of Palaearctic Coleoptera.

[10] Chevrolat L.A.A., Dejean P.F.M.A. (1837). New Taxa. Catalogue des Coléoptères de la Collection de M. le Comte Dejean.

[11] Duponchel P., d’Orbigny C. (1843). Chenesie. Dictionnaire Universel d’Histoire Naturelle Résumant et Complétant Tous les Faits Présentés par les Encyclopédies, les Anciens Dictionnaires Scientifiques, les Oeuvres Complètes de Buffon, et les Meilleurs Traités Spéciaux sur les Diverses Branches des Sciences Naturelles;—Donnant la Descriptiondes Êtres et des Divers Phénomènes de la Nature, l’Étymologie et la Définition des Noms Scientifiques, les Principales Applications des Corps Organiques et Inorganiques à L’agriculture, à la Médecine, aux arts Industriels, etc.; Dirigé par M. Charles d’Orbigny, et Enrichi d’un Magnifique Atlas de Planches Gravées sur Acier.

[12] Montrouzier X. (1855). Essai sur la faune entomologique de l’île de Woodlark ou Moiou. Ann. Des Sci. Phys. Et Nat. D’agriculture Et D’lndustrie Société Lmpériale D’agriculture, etc. de Lyon..

[13] Clark H. (1865). Descriptions of species of phytophaga received from Pulo Penang or its neighbourhood. Ann. Mag. Nat. Hist..

[14] Fairmaire L. (1877). Diagnose de coléoptères de la Nouvelle-Bretagne. Petites Nouv. Entomol..

[15] Weise J., Schenkling S. (1924). Pars 78, Chrysomelidae, 13. Galerucinae. Coleopterorum Catalogus.

[16] Fairmaire L. (1887). Coléoptères des voyages de M. G. Révoil chez les Somalis et dans l’interieur du Zanguebar. Ann. La Société Entomol. Fr..

[17] Baly J.S. (1863). Descriptions of new Phytophaga. Trans. Entomol. Soc. Lond..

[18] Laboissière V. (1919). Descriptions de deux Oides nouveaux, du Tonkin. Bull. La Société Entomol. Fr..

[19] Gressitt J.L., Kimoto S. (1963). The Chrysomelidae (Coleopt.) of China and Korea Part 2. Pac. Insects Monogr..

[20] Laboissière V. (1929). Observations sur les Galerucini asiatiques principalement du Tonkin et du Yunnan et descriptions de nouveaux genres et espèces. Ann. La Société Entomol. Fr..

[21] Kimoto S. (1989). Chrysomelidae (Coleoptera) of Thailand, Cambodia, Laos, and Vietnam. IV. Gallerucinaea. Esakia.

[22] Gahan C.J. (1891). Descriptions of new species of the coleopterous genus *Oides* (Galerucidae). Ann. Mag. Nat. Hist..

[23] Billberg G.J. (1808). New Taxa. Synonymia Insectorum Order: Versuch Einer Synonymie Aller Bisher Bekannten Insecten, Nach Fabricii Systema Eleutheratorum & c. Geordent.

[24] Laboissière V. (1927). Contribution a l’étude des Galerucini de l’Indochine et du Yunnan avec descriptions de nouveaux genres et espès (Col. Chrysomelidae). Ann. Al Société Entomol. Fr..

[25] Roubal J. (1929). Coleoptera nova asiatica. Boll. La Soc. Entomol. Ital..

[26] Roubal J. (1931). Coleopterologische Notizen. Entomol. Nachrichtenblatt.

[27] Jacoby M. (1904). Another contribution to the knowledge of Indian phytophagous Coleoptera. Ann. La Société Entomol. Belg..

[28] Maulik S. (1936). The Fauna of British India, including Ceylon and Burma. Coleoptera. Chrysomelidae (Galerucinae).

[29] Fairmaire L. (1889). Coléptères de l’intérieur de la Chine, 5e partie. Ann. La Société Entomol. Fr..

[30] Weise J. (1922). Chrysomeliden der indo-malayischen Region. Tijdschr. Voor Entomol..

[31] Pic M. (1928). Nouveatués diverses. Mélanges Exot-Entomol..

[32] Samoderzhenkov E.V., Medvedev L.N. (1992). Review of the chrysomelid tribe Luperini (Coleoptera, Chrysomelidae, Galerucinae) from Vietnam. Systematization [sic!] and Ecology of Insects of Vietnam.

[33] Schönherr C.J. (1808). Synonymia Insectorum, Order: Versuch Einer Synonymie Aller Bisher Bekannten Insecten, Nach Fabricii Systema Eleutheratorium Geordnet. Mit Berichtigungen und Anmerkungen, wie auch Beschreibungen Neuer Arten und Illuminirten Kupfern. Erster Band. Eleutherata Oder Käfer. Erster Band. Zweiter Theil. Spercheus-Cryptocephalus.

[34] Guérin-Méneville F.E., Duperrey L.I. (1830). Histoire naturelle des Crustacés, Arachnides et Insectes recueillis dans le voyage autour du Monde de la Corvette de sa Majesté, La Coquille, éxecuté pendant les années 1822, 1823, 1824 et 1825 sous le commendement du capitain Durperrey. Voyang Autour du Monde. Exécuté par Ordre du Roi, sur la Corvette de sa Majesté, La Coquille, Pendant les Années 1822, 1823, 1824 et 1825, sous le Ministère et Conformément aux Instructions de S.E.M. Le Marquies de Clermont-Tonnerre, Minste de la Marine; et Publié sous les Auspices de son Excellence Mgr le Cre de Chabrol, Ministre de la Marine et des Colonies, Zoologie. Vol. 2 (2) Première Division (Crustacées de Insects).

[35] Vachon A. (1980). Galerucinae Oidini de la Nouvelle-Guinée (Col., Chrycomelidae) (3^e^ note) Etudes, synonymies et descriptions d’especes nouvelles. Bull. La Société Entomol. Fr..

[36] Jacoby M. (1891). Descriptions of some new species of phytophagous Coleoptera from India. Enomologist..

[37] Jacoby M. (1899). Descriptions of the new species of phytophagous Coleoptera obtained by Dr. Dohrn in Sumatra. Entomol. Ztg..

[38] Wilcox J.A., Wilcox J.A. (1971). Chrysomelidae: Galerucinae (Oidini, Galerucini, Metacyclini, Sermylini). Coleopterorum Catalogus Supplementa. Pars 78 (1).

[39] Olivier A.G. (1807). Entomologie, ou Histoire Naturelle des Insects, avec Leurs Caratères Génériques et Spécifiques, leur Description, leur Synonymie et Leur Figure Enluminée. Coléoptères. Tome Cinquième.

[40] Fabricius J.C. (1781). Species Insectorum Exhibens Eorum Differentiasm Specificas, Synonyma Auctorum, Loca Natalia, Metamorphosis, Adiectis Observationibus. Tome I.

[41] Pic M. (1927). Coléoptères de l’Indochine. Mélanges Exot.-Entomol..

[42] Hope F.W., Gray J.E. (1831). Synopsis of new species of Nepaul insects in the collection of Major General Hardwicke. Zoological Miscellany.

[43] Chen S.H., Jiang S.Q., Hsieh C.P. (1981). Coleoptera: Chrysomelidae—Galerucinae. The Series of the Scientific Expendition to the Qinghai-Xizang Plateau.

[44] Duvivier A. (1884). Descriptions de quelques phytophages nouveaux. Bull. Ou Comptes-Rendus Des Séances La Société Entomol. Belg..

[45] Jacoby M. (1900). New species of Indian phytophaga principally from Mandar in Bengal. Mémoires La Société Entomol. Belg..

[46] Baly J.S. (1865). Descriptions of new genera and species of phytophaga. Trans. Entomol. Soc. Lond..

[47] Baly J.S. (1879). List of the phytophagous Coleoptera collected in Assam by Chennell, A.W., Esp., with notes and descriptions of the uncharacterized genera and species. Cistula Entomol..

[48] Berti N. (1993). Galerucinae afrotropicaux. Étude des *Oides* Weber, 1801 II. Le groupe *collaris* (Coleoptera, Chrysomelidae). Bull. La Société Entomol. Fr..

[49] Vachon A. (1977). Galerucinae de l’Asie du Sud-Est et de la Nouvelle-Guinée (2^e^ note) [Col. Chrysomelidae] OIDINI.—Nouvelles synonymies. Bull. La Société Entomol. Fr..

[50] Vachon A. (1980). Les Adorium de Fabricius–systématique et synonymies (Col. Chrysomelidae). Bull. La Société Entomol. Fr..

